# The Dynamics of Blood-Count-Derived Inflammatory Indices in the Course of Systemic Treatment for Psoriasis: A Single Center Study

**DOI:** 10.3390/ijms27031612

**Published:** 2026-02-06

**Authors:** Agnieszka Hołdrowicz, Daria Gierach-Michalska, Aleksandra Kośny, Radosław Zajdel, Agnieszka Żebrowska

**Affiliations:** 1 Department of Dermatology and Venereology, Medical University of Lodz, 90-647 Lodz, Poland; 2 Department of Economic and Medical Informatics, University of Lodz, 90-214 Lodz, Poland; 3 Department of Intelligent Systems in Health Monitoring, Medical University of Lodz, 90-645 Lodz, Poland

**Keywords:** psoriasis, biological treatment, methotrexate

## Abstract

Psoriasis is a chronic inflammatory disease that affects up to 3% of the global population. In recent years, monoclonal antibodies targeting key cytokines underlying skin lesions and joint involvement in the course of psoriasis, i.e., TNF-α, IL-17, and IL-23, have been increasingly used due to their high effectiveness and favorable safety profile. Numerous studies have been conducted analyzing the influence of cytokine inhibitors on non-specific inflammatory markers. However, only a limited number of studies on the effect of methotrexate (MTX) therapy on blood-count-derived inflammatory indices in patients with plaque psoriasis have been published so far. The study aims to analyze and compare the impact of methotrexate and biological drugs on the dynamics of selected blood-count-derived inflammatory indices in psoriatic patients. The analysis involved 182 patients receiving biological therapy, which resulted in a total of 219 treatment cycles (TCs) and 48 patients treated with therapeutic doses of MTX (48 TCs). In the biological subgroup, there were six TCs with an inhibitor of IL-12/23, 58 TCs with IL-17A inhibitors, 22 TCs with an inhibitor of IL-17AF, 113 TCs with IL-23 inhibitors, and 20 TCs with TNF-alfa inhibitors. A comparison between patients receiving biological treatment regardless of the drug and patients receiving MTX was conducted. Themajor factors determining the duration of MTX therapy were older age at the time of therapy initiation, a later onset of psoriasis, and a higher burden of comorbidities. Furthermore, the strongest impact on the average inflammatory state over time in patients treated with methotrexate was associated with comorbidities, male gender, and older age. Contrary to MTX therapy, patients receiving biological drugs were characterized by lower values of most assessed blood-count-derived inflammatory biomarkers at week 40 compared to baseline. It was confirmed that biologics and MTX treatment modify the dynamics of blood-count-derived inflammatory biomarkers in a different manner.

## 1. Introduction

Psoriasis (PsO) is a chronic inflammatory disease that affects up to 3% of the global population. However, the incidence rate of psoriasis varies significantly by geographical region [1]. Numerous genetic, epigenetic, immunological, and environmental factors underlie this disease. Psoriasis is mediated by T helper 1 (Th-1), T helper 17 (Th-17), and T helper 22 (Th-22) cells and by cytokines secreted by these cells, and the disruption of the Interleukin-23 (IL-23)/Th17 axis plays a pivotal role in psoriasis pathogenesis. IL-23, released by dendritic cells, stimulates the terminal differentiation of Th-17cells, which produce Interleukin-17 (IL-17), acting as a key effector cytokinein psoriasis. IL-17 not only promotes keratinocyte proliferation but also triggers their abnormal differentiation and the secretion of chemokines and proinflammatory proteins by keratinocytes.

This immune activation is not limited to the skin and leads to a chronic systemic inflammatory state associated with a higher risk of comorbidities, i.e., metabolic syndrome, chronic kidney disease, cardiovascular disorders, and inflammatory bowel disease [1,2,3,4]. Moreover, it has been confirmed that the risk of metabolic syndrome depends on the severity of skin lesions. Higher levels of proinflammatory cytokines in the course of psoriasis causea number of metabolic disorders, i.e., increased insulin resistance, intensified lipogenesis in the liver, and inhibition of lipoprotein lipase activity [5]. Increased levels of Tumor Necrosis Factor-alpha (TNF-α) and interleukin-6(IL-6) in psoriatic patients promote central obesity and insulin resistance. Simultaneously, adipose tissue is a source of various proteins sustaining the chronic inflammatory state, which influences the severity of skin lesions and the effectiveness of treatment [3,4].

In recent years, monoclonal antibodies targeting key cytokines underlying skin lesions and joint involvement in the course of psoriasis, i.e., TNF-α, IL-17, and IL-23, have been increasingly used due to their high effectiveness and favorable safety profile. Therefore, it is of utmost significance to assess the impact of biological drugs for psoriasis on cardiovascular risk and inflammatory markers [6,7,8,9,10]. Numerous studies have been conducted analyzing the influence of cytokine inhibitors on non-specific inflammatory markers [11,12,13,14,15,16,17,18,19,20].

On the other hand, only a limited number of studies on the effect of methotrexate (MTX) therapy on blood-count-derived inflammatory indices in patients with plaque psoriasis have been published so far [21,22,23].

This study provides real-world, longitudinal trajectories of multiple blood-count–derived inflammatory indices in patients with psoriasis treated with MTX or biologic therapies up to week 40, enabling a direct comparison of temporal patterns between conventional systemic therapy and targeted biologics within the same clinical setting. The influence of the severity and localization of skin changes on trajectories of blood morphotic components and blood-count-derived inflammatory biomarkers over time was also assessed. Moreover, factors affecting the effectiveness and duration of methotrexate therapy were evaluated.

## 2. Results

The analysis involved 182 patients receiving biological therapy within the B.47 drug program of the Ministry of Health of the Republic of Poland. Some patients were treated with more than one biological drug, which resulted in a total of 219 treatment cycles (TCs). In this subgroup, there were six TCs with an inhibitor of IL-12/23 (ustekinumab), 58 TCs with IL-17A inhibitors (36 with sekukinumab and 22 with ixekizumab), 22 TCs with an inhibitor of IL-17AF (bimekizumab), 113 TCs with IL-23 inhibitors (70 with risankizumab, 32 with guselkumab, and 11 with tyldrakizumab), and 20 TCs with TNF-alfa inhibitors (18 with adalimumab and two with infliximab). In the analysis, 48 patients were treated with therapeutic doses of MTX (48 TCs). A comparison between patients receiving biological treatment regardless of the drug (BIOL-subgroup) and patients receiving MTX was conducted.

### 2.1. Comparison of Demographic and Clinical Data

There was no statistically significant correlation between sex and receiving MTX or biological drugs. Both among patients treated with MTX and those receiving biological treatment, men predominated, accounting for 60.42% and 56.16% of the respective groups. Patients receiving MTX were statistically significantly older than patients undergoing biological therapies (Mann–Whitney U-test; *p* = 0.0009). The average age of patients treated with MTX was 53.1 ± 17.55 years, and that of patients receiving biologics was 44.11 ± 14.69 years. Moreover, it was observed that patients receiving biologics were characterized by a younger age of disease onset in comparison to those undergoing MTX therapy (Mann–Whitney U-test; *p* = 0.0019).

No statistically significant differences in body weight and Body Mass Index (BMI) values between the two groups were found. There was also no correlation between the severity of skin lesions before treatment initiation and belonging to the BIOL or MTX group.

No statistically significant difference in values of the Charlson Comorbidity Index (CCI) was determined between the two analyzed groups; however, patients treated with MTX appeared to be burdened with more comorbidities than patients receiving biologics.

In both groups, the largest percentage of patients presented disease severity on the Psoriasis Area and Severity Index (PASI) ≥10 at the beginning of treatment (MTX 93.75% vs. 86.76% BIOL). Patients with PASI in the range of 5 to 10 accounted for 2.08% of individuals receiving MTX and 7.31% of patients undergoing biological therapy. Disease severity on the PASI scale ≤ 5 was similar in both groups and amounted to 4.17% of MTX cases and 5.94% of biological patients.

### 2.2. Comparison of Effectiveness of Methotrexate and Biological Therapies

Statistically significantly better effects of biological treatment methods in comparison to MTX were observed from week 16 (+/−4 weeks) of treatment. Percentage-wise, more patients undergoing biological therapies achieved PASI ≤ 5, improving considerably in terms of skin lesions. This observation was maintained at week 40 (+/−4 weeks).

Therapy with MTX resulted in withdrawal statistically significantly more frequently (Fisher’s exact test (RxC); *p* = 0.0023) than biological treatment, both due to adverse reactions (MTX 14.58% vs. 5.05% BIOL) and primary failure (MTX 18.75% vs. 5.96% BIOL). Furthermore, the duration of treatment with MTX was on average shorter than that of biological therapies (Mann–Whitney U-test; *p* = 0.0002). On the other hand, a slightly higher percentage of patients who terminated treatment as a result of a secondary failure was observed in patients treated with biologics (MTX 10.42% vs. 16.51% BIOL).

The clinical and demographic data of the study group are presented in Table 1 and Table 2. The precise statistics, clinical, and demographic data of the study group are presented in Supplementary Tables S1 and S2 in the supplement.

### 2.3. Blood-Count-Derived Inflammatory Biomarkers and Morphotic Blood Components

No statistically significant differences in the initial levels of blood morphotic components, such as leukocytes, neutrophils, lymphocytes, platelets, and monocytes, were found. Analogous results were reported for markers based on the aforementioned parameters. The analysis involved common blood-count-derived inflammatory biomarkers, i.e., Systemic Immune-Inflammation Index (SII), Systemic Inflammation Response Index (SIRI), Aggregate Index of Systemic Inflammation (AISI), Neutrophil-to-Lymphocyte Ratio (NLR), Platelet-to-Lymphocyte Ratio (PLR), Neutrophil-to-Monocyte Ratio (NMR), derived Neutrophil-to-Lymphocyte Ratio (dNLR), Neutrophil-to-Monocyte-to-Lymphocyte Ratio (NMLR), Monocyte-to-Lymphocyte Ratio (MLR). This excluded potential differences and confirmed that the baseline inflammatory state in both patient groups was comparable regardless of the selected treatment method.

After 4 weeks of therapy, a noticeable decrease in the levels of NLR, PLR, dNLR, NMLR, SII, and SIRI markers was reported in individuals receiving biological drugs in comparison to patients treated with MTX, and this observation was statistically significant. An upward trend was observed in patients undergoing methotrexate therapy, after an initial reduction in NLR, dNLR, and SII biomarkers at week 4. In the case of PLR, MLR, NMLR, SIRI, and AISI in individuals receiving MTX, an increase in the values of these parameters was observed at week 4, followed by a decrease at week 16 and a re-increase at week 40. It should be noted that for almost all analyzed biomarkers, the values after 40 weeks of methotrexate treatment were significantly higher than the baseline values. No statistically significant differences were reported between the analyzed groups for the NMR marker at any time point. However, it is worth mentioning that in all patients, NMR increased gradually from week 4. Patients undergoing biological therapies were characterized by lower values of most assessed blood-count-derived inflammatory biomarkers at week 40 compared to baseline. The MLR was the only exception that remained stable throughout the entire observation period.

**Figure 1:**
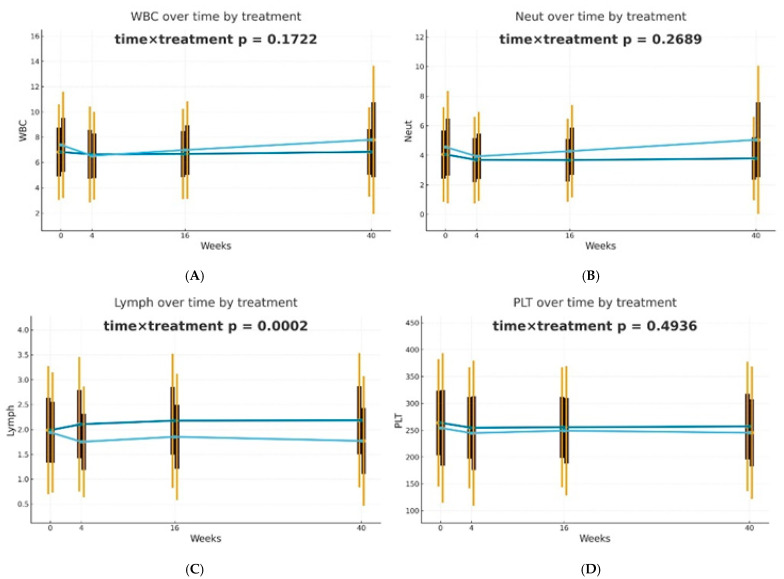

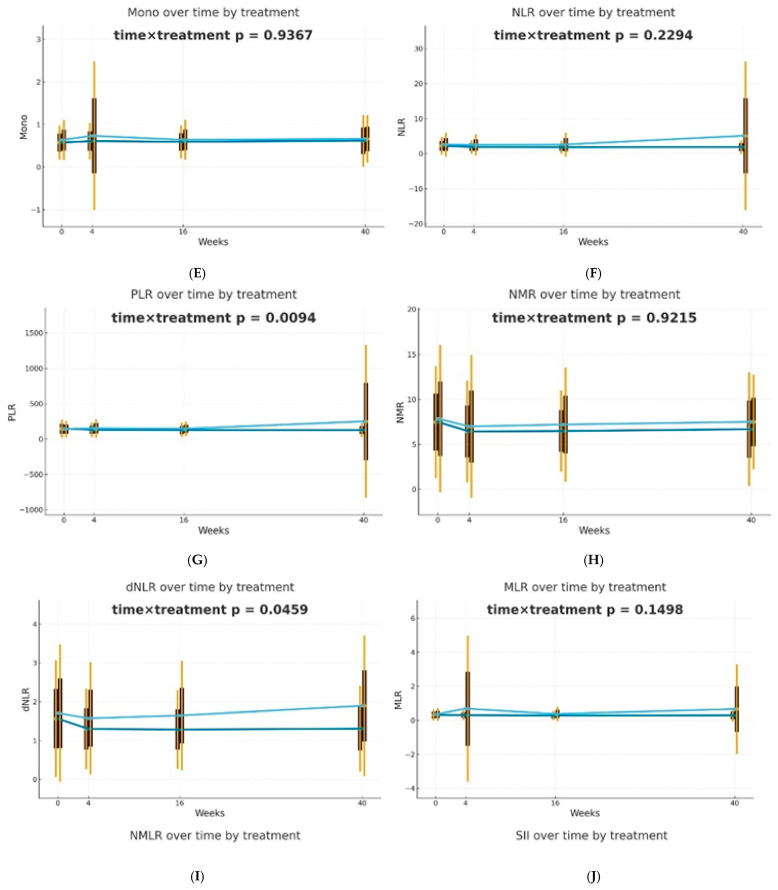
Changes in selected blood morphotic components and blood-count-derived inflammatory biomarkers over time. The figures include the following variables: (**A**) White blood cells (WBC), (**B**) Neutrophils (Neut), (**C**) Lymphocytes (Lymph), (**D**) Platelets (PLT), (**E**) Monocytes (Mono), (**F**) Neutrophil-to-Lymphocyte Ratio (NLR), (**G**) Platelet-to-Lymphocyte Ratio (PLR), (**H**) NMR (Neutrophil-to-Monocyte Ratio), (**I**) dNLR (derived Neutrophil-to-Lymphocyte Ratio), (**J**) MLR (Monocyte-to-Lymphocyte Ratio). Time is represented on the *x*-axis with four marked time points (0/4/16/40 weeks), and measured variables are plotted on the *y*-axis with respective units. Every chart compares both therapeutic approaches (MTX in light blue vs. BIOL in dark blue) for selected parameters. The central points for each time point represent mean values, whereas boxes show the standard deviation around the mean values. Whiskers extend to present the double standard deviation from the mean. Lines connecting central points show changes in mean values over time for the selected treatment method (MTX or BIOL).

Differences in trajectories over time were assessed using Generalized Estimating Equations (GEE) with the omnibus Wald test for the time × treatment interaction as the primary hypothesis. It was observed that biological therapies and MTX treatment modify the dynamics of blood-count-derived inflammatory biomarkers in a different manner. The effectiveness of both therapeutic approaches was confirmed; however, in patients receiving biologics, the inflammatory markers were more stable over time. The decrease in PLR (*p* = 0.0094) and dNLR (*p* = 0.0459) values was statistically significantly more substantial and longer-lasting in biological patients, suggesting a better effect of monoclonal antibodies on suppressing the inflammatory response. No such differences in trajectories between the analyzed groups were reported for NLR, NMLR, SII, SIRI, and AISI, although the average values of these markers decreased gradually for biological patients. The changes over time in the case of most analyzed blood morphotic components, i.e., leukocytes, neutrophils, platelets, and monocytes, were usually milder, leading to insignificant interactions, but confirming the overall similar trend. A reduction in the level of lymphocytes was observed in the group of patients treated with MTX in comparison to biological patients, and this observation was statistically significant (*p* = 0.0002). Trajectories are presented in Figure 1.

**Figure 2:**
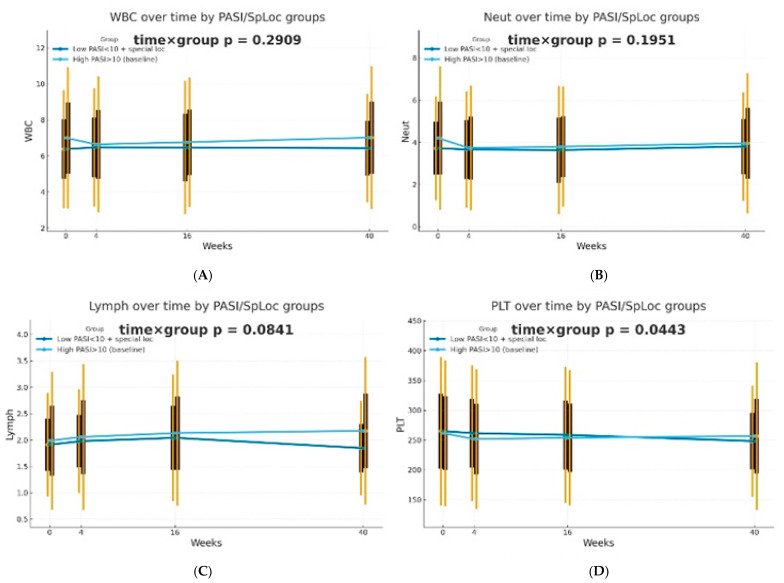

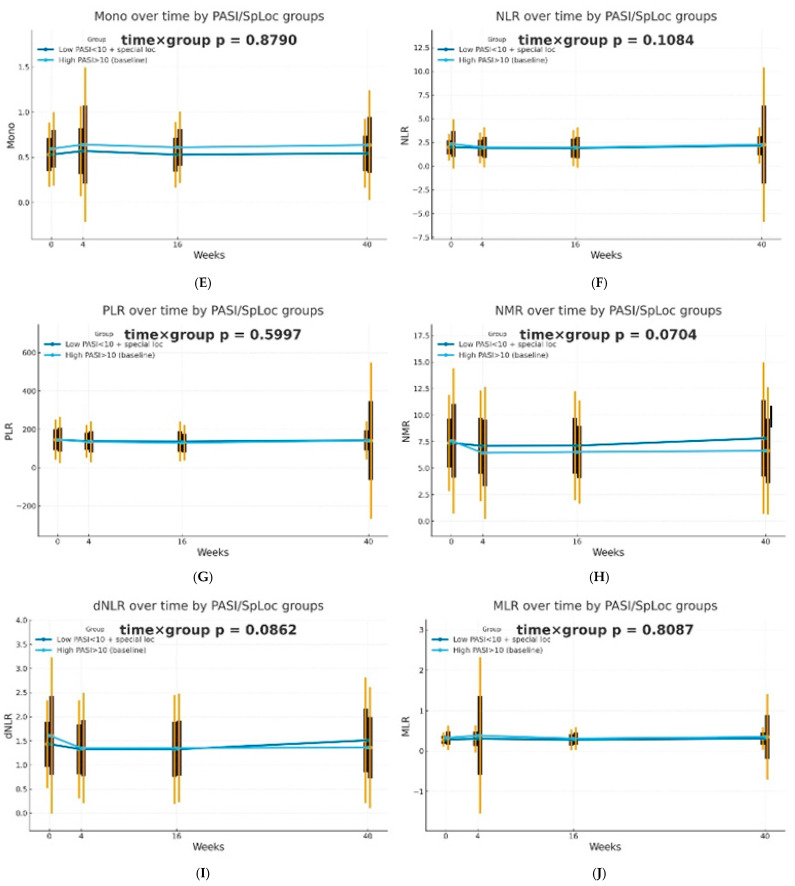

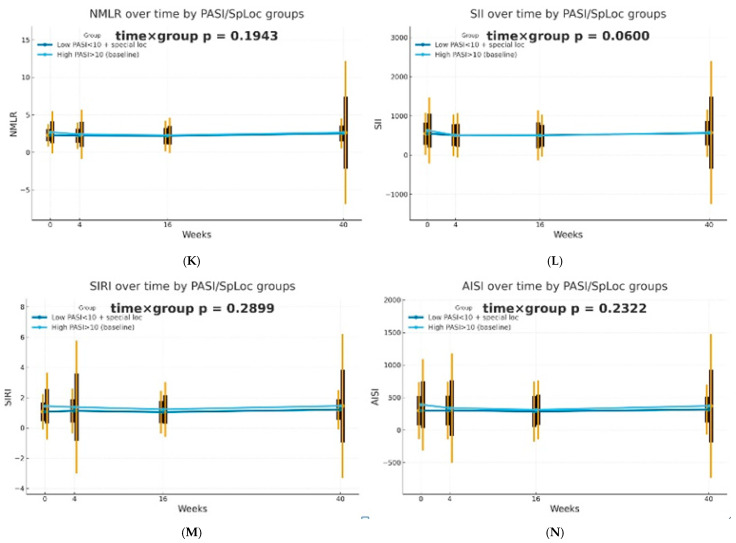
Trajectories for selected blood morphotic components and blood-count-derived inflammatory biomarkers over time for patients characterized by PASI < 10 with lesions in special localizations (dark blue) vs. patients with initial PASI > 10 (light blue); both MTX and BIOL patients were included. The figures include the following variables: (**A**) White blood cells (WBC), (**B**) Neutrophils (Neut), (**C**) Lymphocytes (Lymph), (**D**) Platelets (PLT), (**E**) Monocytes (Mono), (**F**) Neutrophil-to-Lymphocyte Ratio (NLR), (**G**) Platelet-to-Lymphocyte Ratio (PLR), (**H**) NMR (Neutrophil-to-Monocyte Ratio), (**I**) dNLR (derived Neutrophil-to-Lymphocyte Ratio), (**J**) MLR (Monocyte-to-Lymphocyte Ratio), (**K**) Neutrophil-to-Monocyte-to-Lymphocyte Ratio (NMLR), (**L**) Systemic Immune-Inflammation Index (SII), (**M**) Systemic Inflammation Response Index (SIRI), (**N**) Aggregate Index of Systemic Inflammation (AISI).

The dynamics of selected blood morphotic components and blood-count-derived inflammatory biomarkers over time were additionally analyzed using the GEE method (repeated measures at weeks 0/4/16/40, exchangeable working correlation, robust SE) for patients characterized by PASI < 10 with lesions in special localizations and for patients with initial PASI > 10 and compared between both groups. Similar trajectories were obtained for most markers with PLT as one significant exception (interaction time × group: *p* = 0.0443). It is worth mentioning that trends towards diverse trajectories were also observed for SII (*p* = 0.0600), NMR (*p* = 0.0704), Lymph (*p* = 0.0841), and dNLR (*p* = 0.0862). No significant differences in dynamics were determined for other parameters (*p* > 0.10). The results of the analysis are presented in Figure 2.

### 2.4. Factors Affecting the Effectiveness of MTX Therapy

**Figure 3:**
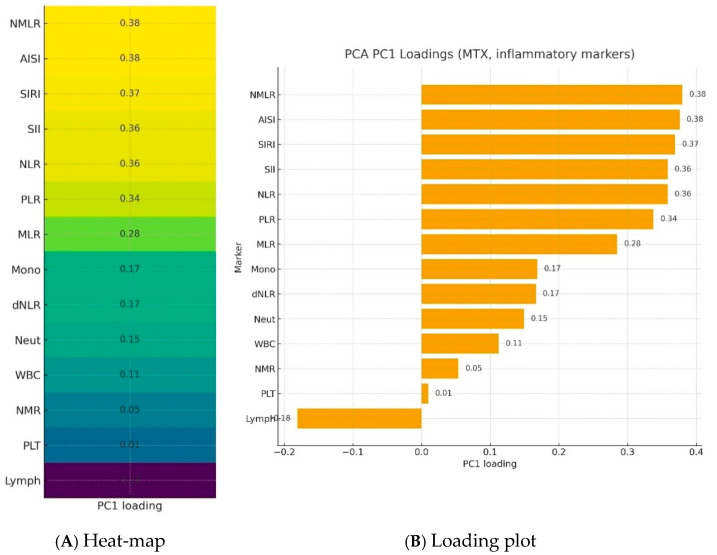
Loadings of blood morphotic components and blood-count-derived inflammatory biomarkers used to create a synthetic variable—Inflammation Index—in a PCA analysis.NMLR—Neutrophil-to-Monocyte-to-Lymphocyte Ratio; AISI—Aggregate Index of Systemic Inflammation; SIRI—Systemic Inflammation Response Index; SII—Systemic Immune-Inflammation Index; NLR—Neutrophil-to-Lymphocyte Ratio; PLR—Platelet-to-Lymphocyte Ratio; MLR—Monocyte-to-Lymphocyte Ratio; Mono—Monocytes; dNLR—derived Neutrophil-to-Lymphocyte Ratio; Neut—neutrophils; WBC—White blood cells; NMR—Neutrophil-to-Monocyte Ratio; Plt—Platelets; Lymph—lymphocytes; PC1—Principal component.

Further analysis took into account a group of patients treated with methotrexate. Blood morphotic components and blood-count-derived inflammatory biomarkers were used to create a synthetic variable (Inflammation Index), which was determined with latent class modeling. The calculated loadings are presented in Figure 3. The most important parameters influencing a latent inflammation index PC1 are NMLR, AISI, SIRI, SII, NLR, and PLR.

Subsequently, a GEE statistical analysis (Gaussian, robust SE) was conducted, in which the predefined latent variable PC1 was modelled as a function of time, covariates, and their interactions (time × covariate). For covariate (Age, MtoT—months to treatment, AoO—age of onset of the disease, weight, BMI, Gender, CCI, PsA) analyses, models of the form PC1_it ~ C(time) × covariate were fit one covariate at a time. The results of the analyses included p(covariate) and p(timexcovariate) and are presented in Table 3.

Based on the performed analysis, it was demonstrated that in the case of patients treated with MTX, comorbidities (CCI), male gender and older age have the strongest impact on the average inflammatory state over time. However, the analyzed factors do not modify the dynamics of PC1 change (p(time × covariate) > 0.22). Regardless of age, gender or comorbidities (CCI), the inflammation rate of change appears similar.

### 2.5. Factors Affecting the Time of Treatment with MTX

**Figure 4:**
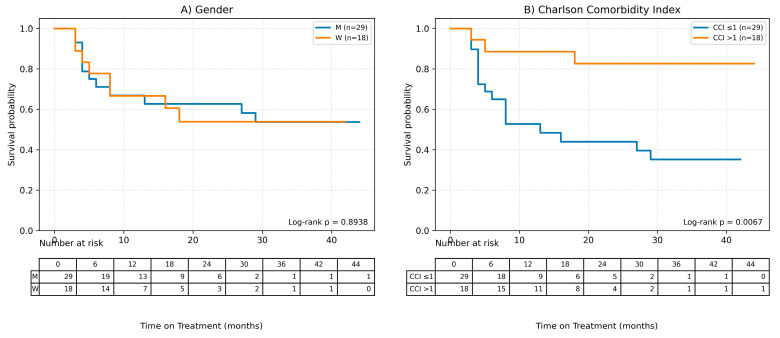

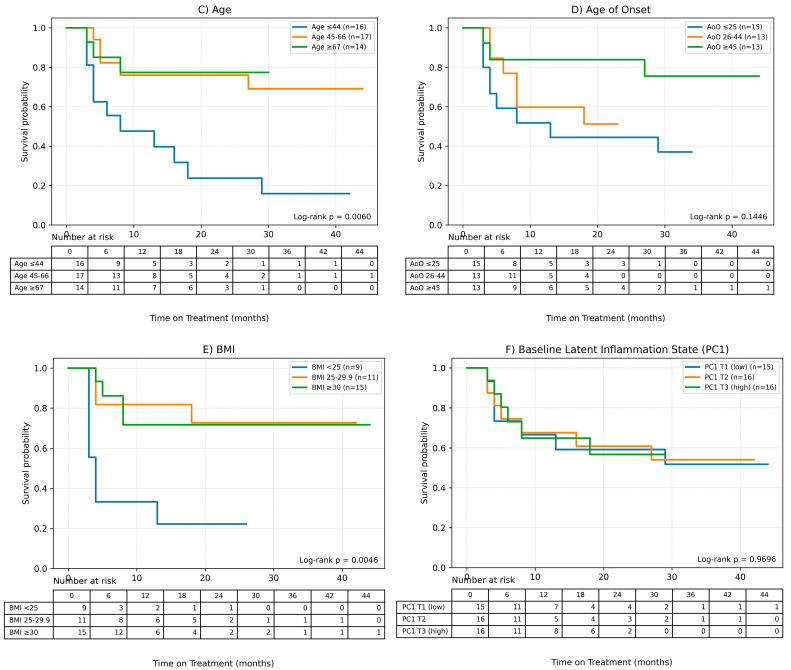
Factors influencing the time of treatment (ToT) in patients treated with MTX. (**A**) Gender, (**B**) Charlson Comorbidity Index, (**C**) Age, (**D**) Age of onset of the disease, (**E**) BMI, (**F**) Baseline Latent Inflammation State PC1.Continuous variables were grouped for visualization purposes as shown below: BMI (WHO): <25; 25–29.9; ≥30. Age (terciles): ≤44.33; 44.33–65.00; >65.00. AoO (terciles): ≤25.00; 25.00–44.00; >44.00. PC1_baseline (terciles): ≤−1.35; −1.35–0.19; >0.19.

Possible factors influencing the time of treatment (ToT) in the case of patients undergoing methotrexate therapy were also assessed in the study using Cox analysis for quantitative variables and Kaplan–Meier survival curves were plotted to analyze qualitative variables. Results of the Cox analysis are presented in Table 4 and Kaplan–Meier curves are shown in Figure 4.

It was observed, based on the conducted Cox analysis, that factors determining shorter MTX treatment duration included older age at the time of therapy initiation (HR = 0.95 (95% CI 0.92–0.98), *p* = 0.0012), later onset of psoriasis (HR = 0.96 (0.93–0.99), *p* = 0.0198) and higher burden of comorbidities according to CCI (HR = 0.54 (0.33–0.89), *p* = 0.0157). On the other hand, disease duration until therapy initiation and baseline latent inflammation state PC1 had no impact on the time of treatment.

The Kaplan–Meier survival curve for CCI shows clear differences between the curves, which is consistent with the results of the Cox analysis—a shorter treatment time is observed for higher CCI values.

## 3. Discussion

Psoriasis, a chronic inflammatory disease, is associated with a number of metabolic complications and is an independent risk factor for cardiovascular disorders [1,2,3,4]. A retrospective registry study conducted on data from Alberta Health Services showed that psoriasis was related to a higher mortality compared to non-psoriatic individuals, and the median age of death was lower than in the general population. Moreover, it was observed that patients with psoriasis were distinguished by a lower average age of death when comparing groups with similar comorbidities according to the CCI [6]. The impact of the treatment applied in the course of psoriasis on the risk of cardiovascular complications and chronic inflammatory state is therefore significant.

Methotrexate is known for its cardioprotective effects, but this characteristic is best documented and evidenced in patients suffering from rheumatoid arthritis [24,25]. The outcomes of in vivo and in vitro research suggest a positive influence of MTX treatment on carbohydrate metabolism, but results are inconsistent [24,26].The methotrexate therapy also seems to be effective in the reduction of endothelial dysfunction in patients with inflammatory arthritis, including psoriatic arthritis [27]; however, findings on the impact of MTX on endothelial function and inflammation are contradictory [24,26,28,29]. In our study, patients receiving MTX were statistically significantly older than patients undergoing biological therapies. This indicates a stronger tendency among dermatologists to treat elderly patients with MTX rather than with biologics. However, in patients with plaque psoriasis, data are scarce and do not clearly confirm the cardioprotective mechanism of methotrexate in this disease [24].

According to the research conducted by Riaz et al., psoriatic patients receiving biological drugs had a lower mortality rate than individuals undergoing MTX therapy or treated with topical medications. The study showed no difference in mortality between patients receiving methotrexate and those subjected to topical therapy, while undergoing any treatment, regardless of method, was associated with better survival compared to non-treated individuals [6].

In a retrospective cohort study, it was observed that patients treated with biologics had lower cardiovascular risk in comparison to patients subjected to conventional systemic therapies (methotrexate, cyclosporin, acitretin, apremilast). Drugs belonging to the groups of TNF-α, IL-17,and IL-23 inhibitors were associated with decreased cardiovascular hazards [9]. Wu et al. determined that individuals receiving TNF-α inhibitors were characterized by lower major cardiovascular event risk compared to patients undergoing MTX therapy [30]. On the other hand, in a meta-analysis involving randomized controlled trials, no difference in risk for Major Adverse Cardiovascular Events (MACE) between biological treatment and placebo was found [31]. A narrative review including cardiovascular imaging studies stated that most published observational clinical studies suggest a protective effect of biological therapies for psoriasis on cardiovascular risk; however, the outcomes of randomized controlled trials assessing primarily vascular inflammation are contradictory. It is worth mentioning that registry data indicate a beneficial impact of MTX and anti-TNF-α therapies on cardiovascular risk [8].

According to the results of our study, the duration of MTX therapy was, on average, shorter than treatment with biological drugs, and an older age at the time of therapy initiation, a later onset of psoriasis, and a higher burden of comorbidities were identified as major factors determining this fact. Furthermore, it was observed that the highest percentage of patients discontinued MTX treatment due to intolerability and primary failure. The strongest impact on the average inflammatory state over time in patients treated with methotrexate was associated with comorbidities, male gender, and older age. Therefore, it seems that in patients burdened with high cardiovascular risk, MTX should be considered as a first-choice therapy with caution. Simultaneously, more effective and tolerable monoclonal antibodies could demonstrate better cardioprotective mechanisms in psoriatic patients.

Numerous population studies reported the relationship between blood-count-derived inflammatory indices and the prevalence of psoriasis [32,33,34]. The association of MLR, NMLR, and SIRI markers with all-cause mortality in psoriatic patients was also determined [32]. Various research indicated a connection between blood-count-derived inflammatory biomarkers and the severity of skin lesions in the PASI score [11,18,19,20,21,23,35,36], but correlations between PASI and NLR, PLR, MPV, or CRP were not confirmed in a retrospective study conducted by Esen [13]. However, Morariu et al. revealed that PSSI is correlated with AISI and SIRI. No relationships were found for the NAPSI score [20].

Most studies reported no associations between blood-count-derived inflammatory indices and PASI score after treatment initiation [11,19,23]; however, Kulakli et al. reported a low statistically significant correlation between PASI change and PLR, SII, and SIRI values after 12 weeks of biological therapy [14]. It was confirmed in our study that the trajectory of platelets over time in the course of psoriasis treatment, for both therapeutic approaches combined, depends on the initial severity of skin lesions. For other parameters, certain trends in trajectories were observed, but without clear statistical significance. In patients with high baseline PASI scores (PASI > 10), a rapid decline followed by a gradual increase in platelet levels was observed. Simultaneously, individuals with low disease severity (PASI < 10) and skin lesions in special localizations were characterized by a slow but steady decline in platelet levels.

Currently, more and more studies are addressing the issue of platelet activation in the pathogenesis of psoriasis. In a study conducted on rats, it was determined that a psoriasis rat model in connection with a blood stasis syndrome (BSS) was characterized by more severe skin lesions examined via hematoxylin and eosin (H&E) staining in comparison to a psoriasis rat model with no blood stasis. In the blood serum of rats with BSS, a higher expression of P-selectin and platelet-activating factor receptor (PAFR) was reported. Clopidogrel administration to rats resulted in a reduction in epidermal thickness, and P2RY12 and GPIIb/IIIa were identified as main targets of clopidogrel, causing a decrease in the severity of psoriatic skin lesions in rats with BSS [37]. Moreover, Merzel Šabović et al. observed the hypercoagulable state due to the impaired fibrinolysis in patients with psoriasis despite effective therapy of skin lesions [38]. Garshick et al. observed an increased activation and aggregation of platelets in patients with successfully treated psoriasis compared to controls. It was also confirmed that the platelet transcriptome in psoriatic individuals is related to proinflammatory and proatherothrombotic pathways [39]. Patients with psoriasis were reported to have an increased activation of platelets in comparison to the control group, and this fact was positively correlated with the severity of skin lesions. Furthermore, platelets of psoriatic patients presented up to three times greater adhesion to aortic endothelial cells [40]. In our study, an increase in the values of PLT was observed at week 4, followed by a decrease at week 16 and a re-increase at week 40 in the group of individuals receiving MTX. Interestingly, in the group of patients treated with biological agents, a substantial decrease in platelet count was observed at week 4, followed by a gradual increase until week 40. In order to draw reliable conclusions, longer observations over time are required. Moreover, it seems that the trajectory of platelets during treatment depends on the initial severity of psoriatic lesions.

Blood-count-derived inflammatory indices represent a chronic low-grade inflammatory state and are used as prognostic biomarkers in patients with cardiovascular diseases [41,42,43]. The summary of numerous studies assessing the impact of biologic therapies on blood-count-derived inflammatory markers conducted so far is presented in Table 5.

Hoffman et al. confirmed a statistically significant reduction in the NLR marker, which then remained stable for 33 months of therapy, in patients treated with both TNF-α inhibitors and ustekinumab. The observed NLR values were lower in patients undergoing adalimumab and etanercept therapies than in patients receiving an inhibitor of IL-12/23 [16]. The dNLR biomarker also declined during treatment with adalimumab between time points measured at months 0, 3, 6, and 12, while the AISI values for certolizumab at these time points varied and did not always decrease, but observed changes were statistically significant. Considerable differences at the abovementioned time points were also reported for other markers and drugs: NLR for ixekizumab, secukinumab, risankizumab, and guselkumab; SIRI for ixekizumab; MLR for secukinumab; AISI for secukinumab and SII for guselkumab. Furthermore, for all monoclonal antibodies combined, PLR, NLR, d-NLR, PMR, SII, and SIRI indices consistently declined throughout the treatment period [20].An et al. also observed that NLR, PLR, MPV, and CRP values were statistically significantly higher before treatment initiation than at 3- and 6-month therapy time points for all assessed biologics (adalimumab, infliximab, etanercept, ustekinumab). No differences were observed between the drugs themselves in this regard [17]. Another retrospective study involving individuals receiving etanercept, adalimumab, and secukinumab confirmed a statistically significant reduction in NLR and PLR values in the course of biological treatment [12]. Moreover, a retrospective study conducted on Japanese patients undergoing infliximab, adalimumab, and ustekinumab therapy also reported a decrease in NLR and PLR values after 12-month treatment [18].

The source literature provides numerous studies analyzing the influence of the two newest groups of biological drugs—IL-17 inhibitors and IL-23 inhibitors—on blood-count-derived inflammatory indices. In a retrospective and observational cohort study, it was determined that treatment with IL-17 inhibitors (secukinumab and ixekizumab) is associated with a statistically significant reduction in NLR, d-NLR, PLR, SII, SIRI, and AISI values after 16 weeks of therapy. No such substantial decrease was observed in patients receiving anti-IL-23 (risankizumab and guselkumab). However, response to therapy in terms of skin lesions was comparable in both medication groups [19].

Kulakli et al. observed a statistically significant greater decrease in PASI score and NLR marker after 12-weektherapy in a group of patients treated with IL-17 inhibitors (secukinumab, ixekizumab) compared to individuals subjected to anti-IL-23 therapies (guselkumab, risankizumab). No substantial differences were observed for other parameters [14]. Another retrospective study assessing the influence of both IL-17 (secukinumab, ixekizumab) and IL-23 (risankizumab, guselkumab) inhibitors on inflammatory state markers reported a statistically significant reduction in NLR, SII, and PHR values after 6 months of treatment, regardless of biological method. No such changes were observed for PLR, RDW, and RPR [11]. In contrast to the results obtained in most research, during biological treatment with IL-17 and IL-23 inhibitors, Esen observed an increase in NLR, PLR, and MPV values with a simultaneous decrease in CRP levels [13].

The majority of available data on the impact of biological therapies on blood-count-derived inflammatory indices comes from retrospective studies based on everyday clinical practice. Post hoc analysis conducted with data from clinical trials VOYAGE I, VOYAGE II, and ECLIPSE confirmed that secukinumab and guselkumab therapies are associated with a statistically significant reduction in NLR, MLR, and PLR values at a similar level. In regard to NLR and PLR, based on VOYAGE I and VOYAGE II data, a greater decrease at week 16 was observed in patients treated with adalimumab than with guselkumab. Furthermore, according to the VOYAGE I clinical trial, adalimumab caused a more substantial decline of MLR in comparison to guselkumab; however, this observation was not confirmed to be statistically significant in the VOYAGE II clinical trial [44].

There is significantly less data on the effect of conventional systemic therapies on blood-count-derived inflammatory indices. Aktaş Karabay et al. observed a statistically significant greater reduction in NLR levels due to adalimumab therapy than methotrexate treatment [23]. In a retrospective study, it was determined that adalimumab, etanercept, and infliximab are more effective in reducing primarily NMR values, but also PLR and NLR markers, compared to methotrexate, acitretin, ustekinumab, secukinumab, and ixekizumab. Furthermore, all abovementioned biologics caused a decrease in NMR levels after 6-month-long treatment, whereas MTX and acitretin induced no such results [15]. Another retrospective study demonstrated that both MTX and biological therapies lead to a reduction in WBC, NLR, and neutrophil levels [21]. In contradiction to the aforementioned results, in our MTX-treated group, we observed a decrease inleukocytes and neutrophils at week 4, followed by a substantial increase to levels higher than baseline.

The results of our study are generally consistent with data published in the source literature. After 4 weeks of therapy, a noticeable decrease in the levels of NLR, PLR, dNLR, NMLR, SII, and SIRI markers was reported in individuals receiving biological drugs in comparison to patients treated with MTX, and this observation was statistically significant. Furthermore, these results were confirmed by better clinical response on the PASI scale from week 16 (+/−4 weeks) in the group of patients treated with biologics. Moreover, it was observed that therapies with biologics and MTX treatment modify the dynamics of blood-count-derived inflammatory biomarkers in a different manner, and in patients receiving biologics, the inflammation markers were more stable over time. The aforementioned results suggested a better effect of monoclonal antibodies on suppressing the inflammatory response.

To the best of our knowledge, there is no other study assessing the impact of MTX therapy on a dNLR biomarker. A statistically significant, more substantial and longer-lasting decrease in dNLR and PLR values was observed in a group of patients receiving biological therapies, indicating the potential value of dNLR as an inflammation biomarker in biological rather than in MTX therapy. A reduction in lymphocyte count was observed only in the group of individuals treated with methotrexate. Şener et al. also reported a decrease in neutrophil count in patients undergoing biological therapies with no change in lymphocyte count [21].

Similar to earlier reports, biologics were more effective in lowering NLR- and PLR-based markers of systemic inflammation. However, in contrast to most available data, our longitudinal analysis revealed a rebound increase of nearly all inflammatory indices during prolonged methotrexate therapy, with values exceeding baseline after 40 weeks, suggesting limited long-term anti-inflammatory control with MTX in plaque psoriasis. Notably, unlike prior studies focused mainly on short observation periods, our extended follow-up demonstrates that biologic therapies provide not only a stronger but also a more stable suppression of systemic inflammation over time. This study provides a real-world, longitudinal, direct comparison of temporal patterns between conventional systemic therapy and targeted biologics within the same setting.

The sustained reduction in dNLR observed exclusively in biologically treated patients represents a novel finding and indicates that dNLR may serve as a sensitive marker for long-term inflammatory control during targeted immunomodulatory therapy.

The main limitations of our study were its retrospective, single-center design and relatively small sample sizes; therefore, extrapolation of the results should be done with caution. No complete cardiovascular risk stratification was conducted due to missing data. Moreover, our study focuses on the trajectory of blood-count-derived inflammatory indices, and its results cannot be directly translated into cardiovascular outcomes. Taking into consideration the limited and highly imbalanced sample sizes for individual biologic drugs, biologics were analyzed as a pooled group in the primary models. An exploratory within-biologics analysis by mechanistic class did not reveal significant heterogeneity in trajectories for key indices, supporting this approach. Patients were treated with different MTX dosing regimens, administered orally or subcutaneously, which could have affected treatment effectiveness and may have impacted the study results. Moreover, individuals receiving methotrexate were older than those undergoing biological therapies, which could also have influenced the results obtained. Due to the retrospective, non-randomized design, residual confounding cannot be fully excluded. Although covariate-adjusted and propensity-based sensitivity analyses yielded consistent results, causal interpretations should be made with caution.

Laboratory data availability decreased at week 40, particularly in the MTX arm, largely due to treatment discontinuation prior to that time point. As a consequence, week-40 comparisons should be interpreted as on-treatment estimates and may be affected by informative dropout. We therefore performed sensitivity analyses restricted to earlier time points and a complete-case analysis. The overall conclusions remained similar, but effect sizes at week 40 should be interpreted cautiously. It would be beneficial to conduct a larger assessment of the influence of individual biological drugs on blood-count-derived inflammatory indices in comparison to methotrexate. Furthermore, a single regimen and one form of drug administration in the case of methotrexate would exclude the possible impact of these parameters on treatment results.

## 4. Materials and Methods

Our single-center retrospective study included patients with moderate-to-severe plaque psoriasis (patients with psoriatic lesion severity on the PASI scale > 10, Body Surface Area (BSA) > 10%, Dermatology Life Quality Index (DLQI) or Children’s Dermatology Life Quality Index (cDLQI) >10 points, or patients with psoriatic lesion severity on the PASI and BSA scales < 10 and lesions in special localizations, i.e., nails, scalp, palms and soles, or anogenital area) who received methotrexate or biological therapy in the Department of Dermatology and Venereology at the Medical University of Lodz from 1 March 2014 to 1 March 2025. Patients treated with MTX received doses ranging from 15 to 20 mg per week, depending on tolerability and effectiveness of the therapy, in oral or subcutaneous form. The research included both patients who received methotrexate as a first conventional systemic therapy for psoriasis and those who were unsuccessfully treated or suffered from adverse reactions during therapies with ciclosporin, acitretin or psoralen plus ultraviolet A (PUVA) therapy. All patients treated with MTX were bio-naïve and had previously received no biological therapy for any reason. Additionally, none of the patients receiving MTX during the analyzed period were treated with combination therapy involving retinoids, other immunosuppressive drugs, or phototherapy.

Patients treated with biological therapies received TNF-α (adalimumab, infliximab, certolizumab pegol), Il-17A (ixekizumab, sekukinumab), IL-17A/F (bimekizumab), Il-12/23 (ustekinumab), and Il-23 (guselkumab, risankizumab, tyldrakizumab) inhibitors.

Patients subjected to biologics were previously unsuccessfully treated with at least two conventional methods (methotrexate, ciclosporin, acitretin, PUVA therapy), had contraindications to conventional systemic treatment, or developed adverse reactions during these therapies. The only exception was children and adolescents who qualified for biological treatment directly after an unsuccessful topical therapy.

A primary failure means that the patient does not reach at least a PASI75 response or a PASI50 response with a simultaneous reduction in the DLQI or cDLQI score by at least 5 points at week 16 (+/−4 weeks) of treatment. In the case of special localizations, worsening of skin changes or no improvement at week 16 (+/−4 weeks) despite therapy determines a primary failure. A secondary failure is defined as an increase in the severity of the disease characterized by PASI > 10, BSA > 10, and DLQI > 10 points or no improvement or worsening of skin changes in patients with lesions in special localizations during two consecutive follow-up appointments.

The following exclusion criteria were defined for this study: active infection at the time of laboratory testing, systemic steroid therapy, pregnancy and lactation period, and other diagnosed diseases with an inflammatory background.

All patients treated were examined in terms of the severity of skin lesions using the PASI, BSA, and DLQI scales and underwent laboratory tests prior to treatment initiation and at weeks 4 (+/−1 week), 16 (+/−4 weeks), and 40 (+/−4 weeks) of therapy. Depending on the severity of psoriatic lesions, patients were divided into three subgroups: PASI ≤ 5, 5 < PASI < 10, and PASI ≥ 10. Patients characterized by PASI < 10 had skin lesions in special localizations, i.e., nails, scalp, palms and soles, or anogenital area. Additionally, the Charlson Comorbidity Index (CCI) was calculated for every patient [45].

Hematological inflammatory parameters were analyzed. Based on the results of laboratory tests, the levels of leukocytes (WBC), neutrophils (Neut), lymphocytes (Lymph), monocytes (Mono), and platelets (PLT) were used to determine and analyze the Systemic Immune-Inflammation Index (SII), Systemic Inflammation Response Index (SIRI), Aggregate Index of Systemic Inflammation (AISI), Neutrophil-to-Lymphocyte Ratio (NLR), Platelet-to-Lymphocyte Ratio (PLR), Neutrophil-to-Monocyte Ratio (NMR), derived Neutrophil-to-Lymphocyte Ratio (dNLR), Neutrophil-to-Monocyte-to-Lymphocyte Ratio (NMLR),and Monocyte-to-Lymphocyte Ratio (MLR) at every time point, i.e., prior to treatment initiation, at week 4 (+/−1 week), 16 (+/−4 weeks), and 40 (+/−4 weeks) of therapy. The definitions of selected blood-count-derived inflammatory markers are presented in Table 6.

This retrospective study relies exclusively on the medical history of patients concerning the severity of psoriatic skin lesions, time of treatment, type of drug, anthropometric data, comorbidities, and laboratory test results. This research was conducted in accordance with the Declaration of Helsinki, Good Clinical Practice rules, and all applicable legal regulations. Patients were provided with psoriasis treatment in line with recommendations of medical associations and clinical indications. The study obtained a positive opinion, No. RNN/142/25/KE, from the Bioethics Committee at the Medical University of Lodz.

### 4.1. Statistical Analysis

#### 4.1.1. Data Structure and Endpoints

Patients underwent repeated laboratory assessments at nominal weeks 0, 4(+/−1), 16(+/−4), and 40(+/−4). For each inflammatory marker (WBC, Neut, Lymph, PLT, Mono, NLR, PLR, NMR, dNLR, MLR, NMLR, SII, SIRI, AISI), values were recorded per time point. The time-to-event endpoint for the MTX arm was Time on Treatment (ToT), with the event defined as any withdrawal status different from “cont”. Observations still on therapy at the last contact were treated as right-censored. Where ties occurred in event times, Breslow handling was applied.

For group definitions, categorical PASI-0 used the provided labels (“≤5”, “5–10”, “≥10”). Special locations were defined by non-missing SpLoc. Unless stated otherwise, analyses were performed in a complete-case fashion per model (no imputation). For repeated-measures models, only rows with non-missing outcome, time, and grouping factor were retained, while for survival analyses, durations and event indicators had to be non-missing.

Due to limited and highly imbalanced sample sizes for individual biologic drugs, biologics were analyzed as a pooled group in the primary models. An exploratory within-biologics analysis by mechanistic class did not reveal significant heterogeneity in trajectories for key indices, supporting this approach.

The Mann–Whitney U-test was used to determine differences between the MTX and BIOL groups. The Chi-squared and Fisher’s exact tests were used to find associations between the subgroups for given variables.

#### 4.1.2. Latent Inflammation Index (PCA)

To obtain an integrative index of systemic inflammation, a first principal component (PC1) was built from the inflammatory markers. Each marker was standardized (z-score), PCA was fit, and the PC1 direction was oriented so that higher PC1 corresponds to a higher inflammatory burden (sign chosen by the sum of loadings) [46]. For descriptive loadings, all available MTX observations across time points were used. For survival models, a baseline PC1 from week-0 markers was employed.

#### 4.1.3. Repeated-Measures Modeling (GEE)

Groupwise differences in trajectories over time were assessed using Generalized Estimating Equations (GEE) with an identity link and Gaussian variance, clustering at the patient level, and an exchangeable working correlation; robust (sandwich) standard errors were used for inference [47,48].

*Model specification: Outcome_it = β_0_ + α_time(t) + γ_group(i) + (αγ)_(time × group) + ε_it.*

For covariate analyses in the MTX arm, models of the form *PC1_it ~ C(time) × covariate* were fit one covariate at a time (Age, MtoT, AoO, weight, BMI, Gender, ChCI, PsA). For PASI/SpLoc comparisons, models of the form *marker_it ~ C(time) × C(group)* were fit separately per marker, where group was: Low PASI < 10 with special locations (PASI-0 in {“≤5”, “5–10”} and SpLoc present) versus High PASI ≥ 10 at baseline. The primary hypothesis in these GEE models was the omnibus Wald test for the *time × group* (or *time × covariate*) *interaction*, which probes differences in dynamics (shape/slope) across groups. A sensitivity fit with independence correlation was used when exchangeable failed to converge.

As sensitivity analyses addressing baseline confounding, we additionally fitted multivariable GEE models adjusted for age, sex, CCI, PsA, baseline PASI category and special locations, and we conducted propensity-score weighted GEE analyses. The primary hypothesis remained the omnibus Wald test for the time×treatment interaction.

Survival analysis (MTX group) was considered useful for further analysis. Kaplan–Meier curves were constructed for prespecified strata: Charlson Comorbidity Index (CCI, median split), gender (F/M), and additionally for PsA (Yes/No), BMI (WHO: <25, 25–29.9, ≥30), age (terciles), age of onset (terciles), and baseline PC1 (terciles) [49]. Two-sample differences were tested with the log-rank test. For k ≥ 3 groups, a k-sample global log-rank test was used [50]. Hazard ratios were estimated using Cox proportional hazards models in a univariable way (one predictor per model) with ties handled by Breslow’s method [51].

Where many markers were screened in parallel (e.g., 14 GEE interaction tests across markers), unadjusted *p*-values were reported alongside the option to apply Holm’s step-down family-wise error control or FDR in confirmatory settings [52].

## 5. Conclusions

The major factors determining the duration of MTX therapy were older age at the time of therapy initiation, a later onset of psoriasis, and a higher burden of comorbidities. Furthermore, it was observed that the highest percentage of patients discontinued MTX treatment due to intolerability and primary failure. The strongest impact on the average inflammatory state over time in patients treated with methotrexate was associated with comorbidities, male gender, and older age. Contrary to MTX therapy, patients receiving biological drugs were characterized by lower values of most assessed blood-count-derived inflammatory biomarkers at week 40 compared to baseline. Biologics and MTX treatment modify the dynamics of blood-count-derived inflammatory biomarkers in a different manner. It was determined that inflammatory indices were more stable over time in biological patients, which suggests a better effect of monoclonal antibodies on suppressing the inflammatory response. The trajectory of platelets in the course of psoriasis treatment, for both therapeutic approaches combined, depended on the initial severity of skin lesions. Further research is required to assess the influence of individual biological drugs on blood-count-derived inflammatory indices in comparison to methotrexate.

## Figures and Tables

**Table 1:** Statistics for quantitative variables for patients treated with methotrexate and biological therapies.

Variable	Minimum	Maximum	Mean	SD
Age (years)				
BIOL	10.00	79.00	44.11	14.69
MTX	15.00	85.00	53.10	17.55
Total	10.00	85.00	45.73	15.60
BMI (kg/m^2^)				
BIOL	15.98	86.96	29.22	7.30
MTX	17.99	44.08	29.86	7.00
Total	15.98	86.96	29.31	7.25
Age of onset (months)				
BIOL	3.00	75.00	25.54	14.01
MTX	5.00	66.00	34.50	17.39
Total	3.00	75.00	26.98	14.93
Duration of disease until treatment initiation (months)				
BIOL	10.00	662.00	224.11	160.48
MTX	12.00	840.00	211.71	175.70
Total	10.00	840.00	222.11	162.73
Time of treatment (months)				
BIOL	3.00	118.00	21.91	16.60
MTX	3.00	44.00	13.57	10.86
Total	3.00	118.00	20.44	16.04

SD—Standard deviation; BIOL—biological treatment; MTX—methotrexate.

**Table 2:** Statistics for qualitative variables with grouping. The Chi-squared and Fisher’s exact tests were used to determine associations between the MTX and BIOL groups for given variables (*p*-value).

Variable	Biological Therapy (% of Total)	Methotrexate (% of Total)	*p*-Value
Gender			0.5900
Men	123 (46.07%)	29 (10.86%)	
Women	96 (35.96%)	19 (7.12%)	
Total	219 (82.02%)	48 (17.98%)	
Drug			<0.001
Adalimumab	18 (6.74%)		
Infliximab	2 (0.75%)		
Guselkumab	32 (11.99%)		
Risankizumab	70 (26.22%)		
Tildrakizumab	11 (4.12%)		
Ustekinumab	6 (2.25%)		
Sekukinumab	36 (13.48%)		
Ixekizumab	22 (8.24%)		
Bimekizumab	22 (8.24%)		
MTX		48 (17.98%)	
Baseline PASI			0.4292
≤5	13 (4.87%)	2 (0.75%)	
5–10	16 (5.99%)	1 (0.37%)	
≥10	190 (71.16%)	45 (16.85%)	
PASI at week 16			<0.001
≤5	98 (36.98%)	12 (4.53%)	
5–10	12 (4.53%)	18 (6.79%)	
≥10	11 (4.15%)	9 (3.40%)	
0	98 (36.98%)	7 (2.64%)	
PASI at week 40			<0.001
≤5	64 (31.84%)	10 (4.98%)	
5–10	3 (1.49%)	7 (3.48%)	
≥10	16 (7.96%)	4 (1.99%)	
0	94 (46.77%)	3 (1.49%)	
Withdrawal			0.0023
AE	11 (4.14%)	7 (2.63%)	
cont	158 (59.40%)	27 (10.15%)	
InP	13 (4.89%)	9 (3.38%)	
InS	36 (13.53%)	5 (1.88%)	
CCI			0.0537
0	96 (35.96%)	18 (6.74%)	
1	50 (18.73%)	12 (4.49%)	
2	46 (17.23%)	9 (3.37%)	
3	15 (5.62%)	2 (0.75%)	
4	6 (2.25%)	2 (0.75%)	
5	6 (2.25%)	1 (0.75%)	
6	0 (0.00%)	1 (0.37%)	
8	0 (0.00%)	1 (0.37%)	
9	0 (0.00%)	1 (0.37%)	
10	0 (0.00%)	1 (0.37%)	

PASI—Psoriasis Area and Severity Index; CCI—Charlson Comorbidity Index.

**Table 3:** Results of the GEE analysis, in which the latent variable PC1 was modeled as a function of time, covariates and their interactions (time × covariate).

Covariate	p_Main_Covariate	p_Interaction_Time × Covariate
Age	0.04	0.62
MtoT	0.66	0.59
AoO	0.18	0.87
weight	0.52	0.23
BMI	0.90	0.68
Gender	<0.01	0.30
CCI	<0.01	0.25
PsA	0.32	0.35

AoO—Age of onset; BMI—Body Mass Index; CCI—Charlson Comorbidity Index; MtoT—Months to treatment; PsA—Psoriatic arthritis.

**Table 4:** Results of the Cox analysis for various possible factors influencing the ToT with MTX.

Variable	coef	SE	z	*p*	HR	HR 95% CI Low	HR 95% CI High
Age	−0.06	0.02	−3.23	<0.01	0.95	0.92	0.98
CCI	−0.62	0.26	−2.42	0.02	0.54	0.33	0.89
BMI	−0.08	0.05	−1.74	0.08	0.92	0.84	1.01
MtoT	−0.001	<0.01	−0.30	0.76	1.00	0.99	1.01
AoO	−0.04	0.02	−2.33	0.02	0.96	0.93	0.99
PC1_baseline	−0.10	0.09	−1.06	0.29	0.91	0.76	1.09

CCI—Charlson Comorbidity Index; BMI—Body Mass Index; MtoT—Months to treatment; AoO—age of onset; PC1_baseline—Baseline Latent Inflammation State.

**Table 5:** The summary of research on the impact of monoclonal antibodies on blood-count-derived inflammatory indices.

Authors	Monoclonal Antibody	Analyzed Indices	Time Points
Çelik MS et al. [11]	IL-23 (risankizumab, guselkumab) or IL-17 inhibitors (secukinumab and ixekizumab)	NLR, PLR, SII, PHR, RPR	0/6 months
Cingöz K et al. [12]	adalimumab, etanercept, infliximab, ustekinumab, or secukinumab	hemoglobin, platelet count, lymphocyte count, neutrophil count, white blood cell count, eosinophil count, monocyte count, basophil count, mean corpuscular volume (MCV), NLR, PLR, MPV, CRP, total cholesterol, high-density lipoprotein (HDL), low-density lipoprotein (LDL), triglycerides, atherogenic index	0/3/6 months
Esen M. [13]	secukinumab, ixekizumab, risankizumab, and guselkumab	NLR, PLR, MPV, and CRP	0/3/6 months
Albayrak H. [15]	adalimumab, etanercept, and infliximab; the IL-17A antagonists ustekinumab, secukinumab, and ixekizumab; and acitretin and methotrexate	NLR, NMR, PLR, and SII	0/3/6 months
Kulakli S et al. [14]	IL-17, IL-23, and IL-12/23 inhibitors	NLR, PLR, MLR, SII, and SIRI	0/3 months
Hoffmann JHO et al. [16]	adalimumab, etanercept, or ustekinumab	NLR	0–990 days
An I [17]	Infliximab, Adalimumab, Etanercept, Ustekinumab	NLR, PLR, MPV	0/3/6 months
Asahina A [18]	infliximab, adalimumab and ustekinumab	NLR, PLR, MPV	0/3/6/12 months
Demirel Öğüt N et al. [19]	secukinumab and ixekizumab, and IL-23 inhibitors (risankizumab and guselkumab)	NLR, d-NLR, PLR, MLR, PMR, SII, SIRI, AISI	0/4 months
Morariu SH et al. [20]	adalimumab etanercept infliximab certolizumab ixekizumab secukinumab tildrakizumab risankizumab guselkumab ustekinumab apremilast	NLR, PLR, PMR, MLR, d-NLR, SIRI, SII, AISI	0/3/6/12 months
Şener G et al. [21]	acitretin, cyclosporine, methotrexate, adalimumab, ustekinumab, risankizumab, guselkumab, secukinumab, ixekizumab	WBC, RBC, MCV, MCH, MCHC, PLT, PCT, neutrophil, lymphocyte, monocytes, basophil, RDW, NLR, MLR, PLR	0/3 months
Merzel Šabović EK et al. [22]	topical therapy, methotrexate, adalimumab, secukinumab or guselkumab	CRP, NLR, PLR, MPR, RPR, complete blood count (CBC) parameters, and disease-specific inflammatory markers	-
AktaşKarabay et al. [23]	narrowband ultraviolet B, acitretin, cyclosporine, methotrexate, adalimumab, etanercept, and ustekinumab	CRP, NLR	0/3 months
Kearneyet al. [44]	guselkumab, sekukinumab, adalimumab	NLR, MLR, PLR, CRP	0/4 months for adalimumab and guselkumab; 0/3 months for sekukinumab andguslekumab

**Table 6:** Definitions of selected blood-count-derived inflammatory markers.

	Definition
NLR	neutrophils/lymphocytes ratio
dNLR	neutrophils/(white blood cells–neutrophils) ratio
MLR	monocytes/lymphocytes ratio
NMLR	(neutrophils + monocytes)/lymphocytes ratio
SIRI	(neutrophils × monocytes)/lymphocytes ratio
SII	(platelets × neutrophils)/lymphocytes ratio
AISI	(neutrophils × monocytes × platelets)/lymphocytes ratio
PLR	platelets/lymphocytes ratio
NMR	neutrophils/monocytes ratio

## Data Availability

The data used in this study are available from the corresponding author upon request.

## Supplementary Materials

The following supporting information can be downloaded at: https://www.mdpi.com/article/10.3390/ijms27031612/s1.

## Abbreviations

The following abbreviations are used in this manuscript:

RxC	Fisher exact
BIOL	group of patients receiving biological treatment
AE	Adverse Effects
AISI	Aggregate Index of Systemic Inflammation
AISI_0	Baseline Aggregate Index of Systemic Inflammation value
AISI_4	Aggregate Index of Systemic Inflammation value at week 4 (±1) of treatment
AISI_16	Aggregate Index of Systemic Inflammation value at week 16 (±4) of treatment
AISI_40	Aggregate Index of Systemic Inflammation value at week 40 (±4) of treatment
Anti-IL-17A	Antagonist of subunit A of interleukin 17
Anti-TNF	Tumor necrosis factor inhibitor
Anti-IL-23	Interleukin 23 antagonist
AoO	age of onset
BMI	Body Mass Index
BSA	Body Surface Area
BSA0	Baseline Body Surface Area
BSA16	Body Surface Area at week 16 (±4) of biological treatment
BSA40	Body Surface Area at week 40 (±4) of biological treatment
CBC	Complete Blood Count
CCI	Charlson Comorbidity Index
cDLQI	Children’s Dermatology Life Quality Index
cont	continuation of treatment
DLQI	Dermatology Life Quality Index
DLQI0	Baseline Dermatology Life Quality Index score
DLQI16	Dermatology Life Quality Index score at week 16 (±4) of biological treatment
DLQI40	Dermatology Life Quality Index scoreat week 40 (±4) of biological treatment
dNLR	derived Neutrophil-to-Lymphocyte Ratio
dNLR_0	Baseline derived Neutrophil-to-Lymphocyte Ratio value
dNLR_4	derived Neutrophil-to-Lymphocyte Ratio value at week 4 (±1) of treatment
dNLR_16	derived Neutrophil-to-Lymphocyte Ratio value at week 16 (±4) of treatment
dNLR_40	derived Neutrophil-to-Lymphocyte Ratio value at week 40 (±4) of treatment
GEE	Generalized Estimating Equations
HDL	High-density lipoprotein
IL-12	Interleukin 12
IL-12/23	Interleukin 12 and 23
IL-17	Interleukin 17
IL-17A	Subunit A of interleukin 17
IL-17AF	Subunit A and F of interleukin 17
IL-22	Interleukin 22
IL-23	Interleukin 23
IL-6	Interleukin 6
IL-12/23i	Interleukin 12 and 23 inhibitor
IL-17Ais	Subunit A of interleukin 17 inhibitors
IL-17AFi	Subunit A and F of interleukin 17 inhibitor
IL-23is	Interleukin 23 inhibitors
TNFis	Tumor Necrosis Factor-alpha inhibitors
InP	Primary failure
InS	Secondary failure
LDL	Low-density lipoprotein
Lymph	Lymphocyte count
lymph_0	Baseline lymphocyte count
lymph_4	Lymphocyte count at week 4 (±1) of treatment
lymph_16	Lymphocyte count at week 16 (±4) of treatment
lymph_40	Lymphocyte count at week 40 (±4) of treatment
MLR	Monocyte-to-Lymphocyte Ratio
MLR_0	Baseline Monocyte-to-Lymphocyte Ratio value
MLR_4	Monocyte-to-Lymphocyte Ratio value at week 4 (±1) of treatment
MLR_16	Monocyte-to-Lymphocyte Ratio value at week 16 (±4) of treatment
MLR_40	Monocyte-to-Lymphocyte Ratio value at week 40 (±4) of treatment
Mono	Monocyte count
mono_0	Baseline monocyte count
mono_4	Monocyte count at week 4 (±1) of treatment
mono_16	Monocyte count at week 16 (±4) of treatment
mono_40	Monocyte count at week 40 (±4) of treatment
MTX	Methotrexate
MtoT	Months to treatment
NLR	Neutrophil-to-Lymphocyte Ratio
NLR_0	Baseline Neutrophil-to-Lymphocyte Ratio value
NLR_4	Neutrophil-to-Lymphocyte Ratio value at week 4 (±1) of treatment
NLR_16	Neutrophil-to-Lymphocyte Ratio value at week 16 (±4) of treatment
NLR_40	Neutrophil-to-Lymphocyte Ratio value at week 40 (±4) of treatment
NMLR	Neutrophil-to-Monocyte-to-Lymphocyte Ratio
NMLR_0	Baseline Neutrophil-to-Monocyte-to-Lymphocyte Ratio value
NMLR_4	Neutrophil-to-Monocyte-to-Lymphocyte Ratio value at week 4 (±1) of treatment
NMLR_16	Neutrophil-to-Monocyte-to-Lymphocyte Ratio value at week 16 (±4) of treatment
NMLR_40	Neutrophil-to-Monocyte-to-Lymphocyte Ratio value at week 40 (±4) of treatment
NMR	Neutrophil-to-Monocyte Ratio
NMR_0	Baseline Neutrophil-to-Monocyte Ratio value
NMR_4	Neutrophil-to-Monocyte Ratio value at week 4 (±1) of treatment
NMR_16	Neutrophil-to-Monocyte Ratio value at week 16 (±4) of treatment
NMR_40	Neutrophil-to-Monocyte Ratio value at week 40 (±4) of treatment
Neut	Neutrophil count
neut_0	Baseline neutrophil count
neut_4	Neutrophil count at week 4 (±1) of treatment
neut_16	Neutrophil count at week 16 (±4) of treatment
neut_40	Neutrophil count at week 40 (±4) of treatment
PAL-FEET	Palmoplantar localization of psoriatic lesions
PASI	Psoriasis Area and Severity Index
PASI_0	Baseline Psoriasis Area and Severity Index
PASI_4	Psoriasis Area and Severity Index score at week 4 (±1) of biological treatment
PASI_16	Psoriasis Area and Severity Index score at week 16 (±4) of biological treatment
PASI_40	Psoriasis Area and Severity Index score at week 40 (±4) of biological treatment
PASI50	50% reduction in psoriatic skin lesions
PASI75	75% reduction in psoriatic skin lesions
PC1	Principal component
PC1_baseline	Baseline Latent Inflammation State
PLR	Platelet-to-Lymphocyte Ratio
PLR_0	Baseline Platelet-to-Lymphocyte Ratio value
PLR_4	Platelet-to-Lymphocyte Ratio value at week 4 (±1) of treatment
PLR_16	Platelet-to-Lymphocyte Ratio value at week 16 (±4) of treatment
PLR_40	Platelet-to-Lymphocyte Ratio value at week 40 (±4) of treatment
PLT	Platelet count
plt_0	Baseline platelet count
plt_4	Platelet count at week 4 (±1) of treatment
plt_16	Platelet count at week 16 (±4) of treatment
plt_40	Platelet count at week 40 (±4) of treatment
PsA	Psoriatic arthritis
PsO	Psoriasis
PUVA	Psoralen Ultra-Violet A
*p*	*p*-value
SII	Systemic Immune-Inflammation Index
SII_0	Baseline Systemic Immune-Inflammation Index value
SII_4	Systemic Immune-Inflammation Index value at week 4 (±1) of treatment
SII_16	Systemic Immune-Inflammation Index value at week 16 (±4) of treatment
SII_40	Systemic Immune-Inflammation Index value at week 40 (±4) of treatment
SIRI	Systemic Inflammation Response Index
SIRI_0	Baseline Systemic Inflammation Response Index value
SIRI_4	Systemic Inflammation Response Index value at week 4 (±1) of treatment
SIRI_16	Systemic Inflammation Response Index value at week 16 (±4) of treatment
SIRI_40	Systemic Inflammation Response Index value at week 40 (±4) of treatment
sp_loc	Special locations
TC	Treatment cycle
Th-1	T helper 1
Th-17	T helper 17
Th-22	T helper 22
TNF-α	Tumor Necrosis Factor-alpha
ToT	Time of treatment
Wbc	White blood cell count
wbc_0	Baseline white blood cell count
wbc_4	White blood cell count at week 4 (±1) of treatment
wbc_16	White blood cell count at week 16 (±4) of treatment
wbc_40	White blood cell count at week 40 (±4) of treatment
IQR	Interquartile range
SD	Standard deviation
