# Supplementary material for: The Dynamics of Blood-Count-Derived Inflammatory Indices in the Course of Systemic Treatment for Psoriasis: A Single Center Study
Source: Int J Mol Sci. 2026 Feb 6;27(3):1612. doi: 10.3390/ijms27031612 (PMC12897763; doi:10.3390/ijms27031612)
Supplement: Supplementary file 1 [file ijms-27-01612-s001.zip › ijms-4099115-supplementary.pdf]

Supplementary Table S1. Statistics for quantitative variables for patients treated with methotrexate and biological therapies. The Mann-Whitney U-test was used to assess whether there is a statistically significant difference between the groups for given variables (p_GROUP).

Variable	By	Level	n	Missing	Mean	Median	SD	IQR	min	max	p_GROUP
**age**	**DRUG-BI**	**Overall**	**267**	**0**	**45.73**	**44**	**15.595**	**22.5**	**10.00**	**85.00**	**0.0009**
		biol	219	0	44.11	42.00	14.688	21.00	10.00	79.00	
		mtx	48	0	53.10	53.50	17.548	28.25	15.00	85.00	
**wbc_0**	**DRUG-BI**	**Overall**	**267**	**0**	**6.93**	**6.8**	**1.909**	**2.48**	**3.75**	**18.21**	**0.0828**
		biol	219	0	6.83	6.74	1.862	2.50	3.5	18.21	
		mtx	48	0	7.39	7.24	2.064	2.88	4.30	13.50	
**neut_0**	**DRUG-BI**	**Overall**	**267**	**0**	**4.15**	**3.90**	**1.637**	**1.94**	**1.60**	**14.00**	**0.0857**
		biol	219	0	4.06	3.90	1.573	1.83	1.60	14.00	
		mtx	48	0	4.56	4.27	1.868	2.50	1.76	10.85	
**lymph_0**	**DRUG-BI**	**Overall**	**267**	**0**	**1.98**	**1.80**	**0.628**	**0.90**	**0.70**	**4.00**	**0.5402**
		biol	219	0	1.99	1.90	0.636	0.85	0.70	4.00	
		mtx	48	0	1.94	1.73	0.595	1.01	0.92	3.04	
**plt_0**	**DRUG-BI**	**Overall**	**267**	**0**	**262.14**	**257.00**	**60.49**	**82.00**	**121.00**	**478.00**	**0.1514**
		biol	219	0	263.85	260.00	58.551	78.00	121.00	416.00	
		mtx	48	0	254.31	240.50	68.791	95.75	146.0	478.00	
**mono_0**	**DRUG-BI**	**Overall**	**267**	**0**	**0.59**	**0.56**	**0.197**	**0.25**	**0.10**	**1.32**	**0.0757**
		biol	219	0	0.58	0.56	0.189	0.24	0.17	1.32	
		mtx	48	0	0.63	0.62	0.223	0.31	0.10	1.06	
**nlr_0**	**DRUG-BI**	**Overall**	**267**	**0**	**2.31**	**2.00**	**1.204**	**1.25**	**0.47**	**8.15**	**0.1705**
		biol	219	0	2.24	2.00	1.100	1.19	0.47	6.54	
		mtx	48	0	2.62	2.20	1.574	1.26	0.62	8.15	
**plr_0**	**DRUG-BI**	**Overall**	**267**	**0**	**145.48**	**135**	**56.951**	**61.12**	**50.33**	**363.33**	**0.6790**
		biol	219	0	146.45	135.88	58.070	61.48	50.57	363.33	
		mtx	48	0	141.04	129.91	51.879	54.21	50.33	304.46	
**nmr_0**	**DRUG-BI**	**Overall**	**267**	**0**	**7.55**	**7.1**	**3.262**	**3.22**	**2.63**	**27.14**	**0.6715**
		biol	219	0	7.48	7.10	3.071	3.27	2.83	27.14	
		mtx	48	0	7.85	7.19	4.044	2.86	2.63	26.40	
**d_nlr_0**	**DRUG-BI**	**Overall**	**267**	**0**	**1.59**	**1.43**	**0.767**	**0.73**	**0.38**	**6.4.**	**0.3957**
		biol	219	0	1.56	1.41	0.741	0.74	0.38	6.40	
		mtx	48	0	1.71	1.47	0.873	0.73	0.48	4.77	
**mlr_0**	**DRUG-BI**	**Overall**	**267**	**0**	**0.32**	**0.29**	**0.136**	**0.16**	**0.07**	**0.89**	**0.1285**
		biol	219	0	0.31	0.28	0.129	0.15	0.08	0.88	
		mtx	48	0	0.35	0.30	0.159	0.22	0.07	0.89	
**nmlr_0**	**DRUG-BI**	**Overall**	**267**	**0**	**2.63**	**2.32**	**1.296**	**1.36**	**0.63**	**9.03**	**0.1835**
		biol	219	0	2.55	2.27	1.184	1.25	0.63	7.10	
		mtx	48	0	2.97	2.53	1.692	1.55	0.85	9.03	
**sii_0**	**DRUG-BI**	**Overall**	**267**	**0**	**619.11**	**506.82**	**396.142**	**410.46**	**118.78**	**2835.44**	**0.5880**
		biol	219	0	604.51	492.00	361.577	412.78	118.78	2366.00	
		mtx	48	0	685.74	544.25	525.569	392.74	136.62	2835.44	
**siri_0**	**DRUG-BI**	**Overall**	**267**	**0**	**1.4**	**1.14**	**1.036**	**0.96**	**0.18**	**8.31**	**0.0846**
		biol	219	0	1.32	1.12	0.868	0.88	0.19	5.25	
		mtx	48	0	1.77	1.37	1.552	1.27	0.18	8.31	
**aisi_0**	**DRUG-BI**	**Overall**	**267**	**0**	**379.24**	**285.36**	**333.124**	**266.22**	**34.85**	**2892.15**	**0.3183**
		biol	219	0	357.13	276.85	270.483	245.68	34.85	1703.52	
		mtx	48	0	480.12	322.41	525.394	382.53	43.12	2892.15	
**wbc_4**	**DRUG-BI**	**Overall**	**251**	**16**	**6.62**	**6.37**	**1.833**	**2.08**	**3.2**	**16.16**	**0.8622**
		biol	209	10	6.64	6.37	1.862	2.03	3.20	16.16	
		mtx	42	6	6.53	6.38	1.702	2.24	3.60	10.39	
**neut_4**	**DRUG-BI**	**Overall**	**251**	**16**	**3.73**	**3.50**	**1.44**	**1.77**	**1.47**	**10.20**	**0.2903**
		biol	209	10	3.68	3.46	1.434	1.76	1.47	10.20	
		mtx	42	6	3.93	3.75	1.472	1.93	1.76	7.61	
**lymph_4**	**DRUG-BI**	**Overall**	**251**	**16**	**2.05**	**1.91**	**0.662**	**0.88**	**0.43**	**4.76**	**0.0015**
		biol	209	10	2.11	2.00	0.668	0.88	0.66	4.76	
		mtx	42	6	1.75	1.62	0.547	0.66	0.43	2.86	
**plt_4**	**DRUG-BI**	**Overall**	**251**	**16**	**253**	**247**	**57.651**	**89.5**	**144**	**461**	**0.1557**
		biol	209	10	254.66	252.00	55.643	89.00	144.00	408.00	
		mtx	42	6	244.74	223.50	66.891	80.50	144.00	461.00	
**mono_4**	**DRUG-BI**	**Overall**	**242**	**25**	**0.63**	**0.58**	**0.404**	**0.25**	**0.13**	**6.06**	**0.7052**
		biol	200	19	0.61	0.58	0.206	0.24	0.13	1.48	
		mtx	42	6	0.74	0.59	0.861	0.27	0.27	6.06	
**nlr_4**	**DRUG-BI**	**Overall**	**251**	**16**	**1.99**	**1.77**	**0.976**	**1.06**	**0.43**	**7.88**	**0.0038**
		biol	209	10	1.89	1.72	0.838	1.04	0.43	4.77	

		mtx	42	6	2.50	2.12	1.385	1.24	0.62	7.88	
**plr_4**	**DRUG-BI**	**Overall**	**251**	**16**	**135.22**	**130**	**49.445**	**61.08**	**47.94**	**413.95**	**0.0200**
		biol	209	10	131.78	123.81	46.506	59.66	47.94	312.12	
		mtx	42	6	152.35	148.57	59.787	65.31	51.75	413.95	
**nmr_4**	**DRUG-BI**	**Overall**	**242**	**25**	**6.52**	**6.15**	**3.009**	**2.92**	**0.36**	**31.54**	**0.3406**
		biol	200	19	6.42	6.12	2.784	2.69	1.01	31.54	
		mtx	42	6	6.98	6.61	3.916	3.96	0.36	25.78	
**d_nlr_4**	**DRUG-BI**	**Overall**	**251**	**16**	**1.35**	**1.28**	**0.554**	**0.65**	**0.34**	**4.04**	**0.0142**
		biol	209	10	1.30	1.23	0.507	0.66	0.34	2.93	
		mtx	42	6	1.58	1.46	0.712	0.57	0.48	4.04	
**mlr_4**	**DRUG-BI**	**Overall**	**242**	**25**	**0.37**	**0.29**	**0.894**	**0.15**	**0.09**	**14.09**	**0.0079**
		biol	200	19	0.31	0.29	0.115	0.14	0.09	0.83	
		mtx	42	6	0.69	0.34	2.123	0.16	0.13	14.09	
**nmlr_4**	**DRUG-BI**	**Overall**	**242**	**25**	**2.38**	**2.09**	**1.503**	**1.19**	**0.57**	**19.14**	**0.0028**
		biol	200	19	2.20	2.00	0.919	1.24	0.57	5.48	
		mtx	42	6	3.19	2.49	2.890	1.33	0.85	19.14	
**sii_4**	**DRUG-BI**	**Overall**	**251**	**16**	**504.99**	**462.23**	**269.633**	**362.52**	**88.41**	**1862.59**	**0.0300**
		biol	209	10	486.05	460.14	252.697	317.93	88.41	1375.60	
		mtx	42	6	599.21	506.33	329.287	406.73	136.62	1862.59	
**siri_4**	**DRUG-BI**	**Overall**	**242**	**25**	**1.35**	**1.06**	**2.037**	**0.82**	**0.21**	**30.58**	**0.0220**
		biol	200	19	1.17	1.02	0.697	0.80	0.21	4.06	
		mtx	42	6	2.18	1.26	4.602	0.98	0.41	30.58	
**aisi_4**	**DRUG-BI**	**Overall**	**242**	**25**	**334.71**	**260.19**	**394.716**	**240.74**	**41.2**	**5443.57**	**0.0922**
		biol	200	19	302.97	244.83	200.512	228.85	41.20	1141.75	
		mtx	42	6	485.82	296.38	832.082	287.04	91.53	5443.57	
**wbc_16**	**DRUG-BI**	**Overall**	**265**	**2**	**6.73**	**6.42**	**1.778**	**1.93**	**3.5**	**13.65**	**0.3107**
		biol	219	0	6.67	6.35	1.751	1.85	3.72	13.65	
		mtx	46	2	6.99	6.84	1.899	2.62	3.50	11.10	
**neut_16**	**DRUG-BI**	**Overall**	**264**	**3**	**3.78**	**3.6**	**1.416**	**1.65**	**1**	**9.3**	**0.0080**
		biol	219	0	3.67	3.50	1.372	1.40	1.00	9.30	
		mtx	45	3	4.28	4.02	1.533	2.19	1.65	8.55	
**lymph_16**	**DRUG-BI**	**Overall**	**264**	**3**	**2.12**	**2**	**0.669**	**0.8**	**0.61**	**5.2**	**0.0010**
		biol	219	0	2.18	2.10	0.665	0.80	0.90	5.20	
		mtx	45	3	1.85	1.74	0.625	0.66	0.61	3.49	
**plt_16**	**DRUG-BI**	**Overall**	**265**	**2**	**254.55**	**248**	**55.891**	**73**	**140**	**455**	**0.2866**
		biol	219	0	255.68	252.00	55.185	73.00	140.00	455.00	
		mtx	46	2	249.17	238.00	59.479	64.25	148.00	390.00	
**mono_16**	**DRUG-BI**	**Overall**	**265**	**2**	**0.6**	**0.58**	**0.192**	**0.26**	**0.2**	**1.42**	**0.2176**
		biol	219	0	0.59	0.57	0.184	0.25	0.24	1.42	
		mtx	46	2	0.64	0.61	0.225	0.29	0.20	1.29	
**nlr_16**	**DRUG-BI**	**Overall**	**265**	**2**	**1.95**	**1.78**	**1.01**	**1.08**	**0.33**	**10.82**	**0.0001**
		biol	219	0	1.82	1.71	0.778	0.99	0.33	5.47	
		mtx	46	2	2.59	2.34	1.598	1.28	0.79	10.82	
**plr_16**	**DRUG-BI**	**Overall**	**265**	**2**	**130.4**	**124.44**	**44.705**	**55.96**	**51.21**	**305**	**0.0034**
		biol	219	0	127.22	122.14	44.363	52.63	51.21	305.00	
		mtx	46	2	145.57	146.49	43.658	49.96	56.49	242.62	
**nmr_16**	**DRUG-BI**	**Overall**	**265**	**2**	**6.59**	**6.23**	**2.409**	**2.87**	**1.82**	**19.55**	**0.1505**
		biol	219	0	6.47	6.12	2.219	2.67	1.82	14.33	
		mtx	46	2	7.19	6.95	3.119	3.39	2.49	19.55	
**d_nlr_16**	**DRUG-BI**	**Overall**	**265**	**2**	**1.35**	**1.29**	**0.551**	**0.63**	**0.27**	**4.07**	**0.0003**
		biol	219	0	1.29	1.25	0.497	0.57	0.27	3.64	
		mtx	46	2	1.65	1.52	0.692	0.74	0.56	4.07	
**mlr_16**	**DRUG-BI**	**Overall**	**265**	**2**	**0.3**	**0.28**	**0.128**	**0.14**	**0.09**	**1.23**	**0.0002**
		biol	219	0	0.29	0.27	0.107	0.12	0.09	0.71	
		mtx	46	2	0.38	0.34	0.183	0.18	0.14	1.23	
**nmlr_16**	**DRUG-BI**	**Overall**	**265**	**2**	**2.26**	**2.05**	**1.106**	**1.18**	**0.43**	**12.05**	**0.0001**
		biol	219	0	2.11	1.98	0.851	1.13	0.43	5.87	
		mtx	46	2	2.96	2.69	1.745	1.39	1.11	12.05	
**sii_16**	**DRUG-BI**	**Overall**	**265**	**2**	**496.56**	**445.48**	**263.253**	**315.55**	**66**	**1799.82**	**0.0011**
		biol	219	0	469.40	429.86	241.478	301.98	66.00	1799.82	
		mtx	46	2	625.87	551.00	321.560	442.79	155.63	1639.73	
**siri_16**	**DRUG-BI**	**Overall**	**265**	**2**	**1.21**	**1**	**0.859**	**0.79**	**0.09**	**8.11**	**0.0008**
		biol	219	0	1.10	0.96	0.673	0.72	0.09	4.71	
		mtx	46	2	1.70	1.31	1.353	1.31	0.46	8.11	
**aisi_16**	**DRUG-BI**	**Overall**	**265**	**2**	**308.19**	**237.17**	**220.215**	**224.91**	**18.48**	**1360.97**	**0.0029**
		biol	219	0	286.13	226.92	197.227	165.84	18.48	1223.88	
		mtx	46	2	413.20	335.51	287.087	313.62	106.83	1360.97	
**wbc_40**	**DRUG-BI**	**Overall**	**200**	**67**	**6.95**	**6.66**	**1.919**	**2.43**	**3.79**	**13.47**	**0.2862**
		biol	177	42	6.84	6.60	1.734	2.29	3.79	11.77	

		mtx	23	25	7.79	7.00	2.899	3.98	4.34	13.47	
**neut_40**	**DRUG-BI**	**Overall**	**201**	**66**	**3.94**	**3.7**	**1.597**	**2**	**1.3**	**9.84**	**0.0278**
		biol	177	42	3.79	3.70	1.380	1.90	1.30	8.30	
		mtx	24	24	5.05	4.34	2.478	2.74	2.12	9.84	
**lymph_40**	**DRUG-BI**	**Overall**	**201**	**66**	**2.13**	**2.1**	**0.675**	**0.94**	**0.08**	**5.3**	**0.0127**
		biol	177	42	2.18	2.10	0.665	0.90	1.10	5.30	
		mtx	24	24	1.77	1.75	0.643	0.73	0.08	2.89	
**plt_40**	**DRUG-BI**	**Overall**	**201**	**66**	**255.76**	**253**	**59.614**	**79**	**106**	**462**	**0.2724**
		biol	177	42	257.16	254.00	59.466	78.00	106.00	462.00	
		mtx	24	24	245.42	232.00	60.965	70.50	147.00	382.00	
**mono_40**	**DRUG-BI**	**Overall**	**184**	**83**	**0.62**	**0.57**	**0.289**	**0.26**	**0.13**	**3.2**	**0.3827**
		biol	161	58	0.62	0.57	0.292	0.25	0.13	3.20	
		mtx	23	25	0.66	0.66	0.268	0.27	0.36	1.58	
**nlr_40**	**DRUG-BI**	**Overall**	**201**	**66**	**2.26**	**1.81**	**3.79**	**1.1**	**0.47**	**53.5**	**0.0006**
		biol	177	42	1.87	1.76	0.856	1.04	0.47	5.19	
		mtx	24	24	5.11	2.48	10.473	2.46	1.20	53.50	
**plr_40**	**DRUG-BI**	**Overall**	**201**	**66**	**141.29**	**124.09**	**189.661**	**52.69**	**32.12**	**2750**	**0.0177**
		biol	177	42	126.60	119.55	42.807	49.01	32.12	277.50	
		mtx	24	24	249.64	148.38	533.723	48.01	60.52	2750.00	
**nmr_40**	**DRUG-BI**	**Overall**	**184**	**83**	**6.78**	**6.15**	**3.05**	**3.22**	**1.02**	**24.29**	**0.0756**
		biol	161	58	6.68	6.04	3.105	2.94	1.02	24.29	
		mtx	23	25	7.49	8.23	2.581	4.41	3.27	11.92	
**d_nlr_40**	**DRUG-BI**	**Overall**	**201**	**66**	**1.38**	**1.3**	**0.62**	**0.72**	**0.36**	**4.08**	**0.0010**
		biol	177	42	1.31	1.21	0.540	0.63	0.36	3.45	
		mtx	24	24	1.90	1.71	0.894	1.15	0.88	4.08	
**mlr_40**	**DRUG-BI**	**Overall**	**184**	**83**	**0.34**	**0.27**	**0.486**	**0.14**	**0.09**	**6.5**	**0.0019**
		biol	161	58	0.30	0.27	0.163	0.13	0.09	1.90	
		mtx	23	25	0.66	0.35	1.285	0.17	0.18	6.50	
**nmlr_40**	**DRUG-BI**	**Overall**	**184**	**83**	**2.62**	**2.09**	**4.413**	**1.2**	**0.62**	**60**	**0.0009**
		biol	161	58	2.16	2.02	0.918	1.19	0.62	5.69	
		mtx	23	25	5.81	2.50	11.984	2.46	1.43	60.00	
**sii_40**	**DRUG-BI**	**Overall**	**201**	**66**	**572.38**	**446.06**	**850.422**	**353.86**	**111**	**11770**	**0.0054**
		biol	177	42	488.87	438.96	270.639	340.28	111.00	1799.37	
		mtx	24	24	1188.21	624.65	2297.599	725.43	269.82	11770.00	
**siri_40**	**DRUG-BI**	**Overall**	**184**	**83**	**1.42**	**0.99**	**2.21**	**0.89**	**0.16**	**27.82**	**0.0047**
		biol	161	58	1.17	0.97	0.835	0.77	0.16	7.24	
		mtx	23	25	3.24	1.16	5.624	3.24	0.47	27.82	
**aisi_40**	**DRUG-BI**	**Overall**	**184**	**83**	**363.86**	**254.52**	**515.946**	**246.56**	**35.52**	**6120.4**	**0.0176**
		biol	161	58	307.00	252.67	250.879	243.44	35.52	2069.27	
		mtx	23	25	761.87	296.26	1251.913	513.32	105.23	6120.40	
**MtoT**	**DRUG-BI**	**Overall**	**261**	**6**	**222.11**	**172**	**162.73**	**228**	**10**	**840**	**0.3565**
		biol	219	0	224.11	180.00	160.475	204.00	10.00	662.00	
		mtx	42	6	211.71	138.00	175.701	249.00	12.00	840.00	
**weight**	**DRUG-BI**	**Overall**	**256**	**11**	**86.91**	**85.5**	**21.626**	**26.5**	**35**	**159**	**0.4862**
		biol	219	0	86.54	85.00	21.473	25.50	35.00	155.00	
		mtx	37	11	89.06	87.00	22.692	25.00	46.80	159.00	
**height**	**DRUG-BI**	**Overall**	**255**	**12**	**172.07**	**173**	**10.43**	**15**	**115**	**193**	**1.0000**
		biol	219	0	172.02	173.00	10.570	14.00	115.00	193.00	
		mtx	36	12	172.42	173.00	9.661	15.00	152.00	192.00	
**bmi**	**DRUG-BI**	**Overall**	**255**	**12**	**29.31**	**28.63**	**7.245**	**7.15**	**15.98**	**86.96**	**0.4810**
		biol	219	0	29.22	28.31	7.297	7.13	15.98	86.96	
		mtx	36	12	29.86	29.50	6.996	8.40	17.99	44.08	
**ToT**	**DRUG-BI**	**Overall**	**266**	**1**	**20.44**	**16**	**16.041**	**19.75**	**3**	**118**	**0.0002**
		biol	219	0	21.91	18.00	16.600	21.50	3.00	118.00	
		mtx	47	1	13.57	10.00	10.858	15.50	3.00	44.00	
**AoO**	**DRUG-BI**	**Overall**	**261**	**6**	**26.98**	**25**	**14.933**	**15**	**3**	**75**	**0.0019**
		biol	219	0	25.54	23.00	14.005	15.00	3.00	75.00	
		mtx	42	6	34.50	32.00	17.388	28.00	5.00	66.00	

AISI_0 - Baseline Aggregate Index of Systemic Inflammation value; AISI_4 - Aggregate Index of Systemic Inflammation value at week 4 (±1) of treatment; AISI_16 - Aggregate Index of Systemic Inflammation value at week 16 (±4) of treatment; AISI_40 - Aggregate Index of Systemic Inflammation value at week 40 (±4) of treatment; AoO - age of onset; BMI - Body Mass Index; BIOL Biological treatment; dNLR_0 - Baseline derived Neutrophil-to-Lymphocyte Ratio value; dNLR_4 - derived Neutrophil-to-Lymphocyte Ratio value at week 4 (±1) of treatment; dNLR_16 - derived Neutrophil-to-Lymphocyte Ratio value at week 16 (±4) of treatment; dNLR_40 - derived Neutrophil-to Lymphocyte Ratio value at week 40 (±4) of treatment; lymph_0 - Baseline lymphocyte count; lymph_4 - Lymphocyte count at week 4 (±1) of treatment; lymph_16 - Lymphocyte count at week 16 (±4) of treatment; lymph_40 - Lymphocyte count at week 40 (±4) of treatment; MLR_0 - Baseline Monocyte-to-Lymphocyte Ratio

value; MLR_4 - Monocyte-to-Lymphocyte Ratio value at week 4 (±1) of treatment; MLR_16 - Monocyte-to-Lymphocyte Ratio value at week 16 (±4) of treatment; MLR_40 - Monocyte-to-Lymphocyte Ratio value at week 40 (±4) of treatment; mono_0 - Baseline monocyte Mount; mono_4 - Monocyte count at week 4 (±1) of treatment; mono_16 - Monocyte count at week 16 (±4) of treatment; mono_40 - Monocyte count at week 40 (±4) of treatment; MTX - Methotrexate; MtoT - Months to treatment; NLR_0 - Baseline Neutrophil-to-Lymphocyte Ratio value; NLR_4 - Neutrophil-to-Lymphocyte Ratio value at week 4 (±1) of treatment; NLR_16 - Neutrophil-to-Lymphocyte Ratio value at week 16 (±4) of treatment; NLR_40 - Neutrophil-to-Lymphocyte Ratio value at week 40 (±4) of treatment; NMLR_0 - Baseline Neutrophil-to-Monocyte-to-Lymphocyte Ratio value; NMLR_4 - Neutrophil-to Monocyte-to-Lymphocyte Ratio value at week 4 (±1) of treatment; NMLR_16 - Neutrophil-to-Monocyte-to-Lymphocyte Ratio value at week 16 (±4) of treatment; NMLR_40 - Neutrophil-to-Monocyte-to-Lymphocyte Ratio value at week 40 (±4) of treatment; NMR_0 - Baseline Neutrophil-to-Monocyte Ratio value; NMR_4 - Neutrophil-to-Monocyte Ratio value at week 4 (±1) of treatment; NMR_16 - Neutrophil-to-Monocyte Ratio value at week 16 (±4) of treatment; NMR_40 - Neutrophil-to-Monocyte Ratio value at week 40 (±4) of treatment; neut_0 - Baseline neutrophil count; neut_4 - Neutrophil count at week 4 (±1) of treatment; neut_16 - Neutrophil count at week 16 (±4) of treatment; neut_40 - Neutrophil count at week 40 (±4) of treatment; PLR_0 - Baseline Platelet-to-Lymphocyte Ratio value; PLR_4 - Platelet-to-Lymphocyte Ratio value at week 4 (±1) of treatment; PLR_16 - Platelet-to-Lymphocyte Ratio value at week 16 (±4) of treatment; PLR_40 - Platelet-to-Lymphocyte Ratio value at week 40 (±4) of treatment; plt_0 - Baseline platelet Mount; plt_4 - Platelet count at week 4 (±1) of treatment; plt_16 - Platelet count at week 16 (±4) of treatment; plt_40 - Platelet count at week 40 (±4) of treatment; SII - Systemic Immune-Inflammation Index; SII_0 - Baseline Systemic Immune-Inflammation Index value; SII_4 - Systemic Immune-Inflammation Index value at week 4 (±1) of treatment; SII_16 - Systemic Immune-Inflammation Index value at week 16 (±4) of treatment; SII_40 - Systemic Immune-Inflammation Index value at week 40 (±4) of treatment; SIRI_0 - Baseline Systemic Inflammation Response Index value; SIRI_4 - Systemic Inflammation Response Index value at week 4 (±1) of treatment; SIRI_16 - Systemic Inflammation Response Index value at week 16 (±4) of treatment; SIRI_40 - Systemic Inflammation Response Index value at week 40 (±4) of treatment; ToT - Time of treatment; wbc_0 - Baseline white blood cell Mount; wbc_4 - White blood cell count at week 4 (±1) of treatment; wbc_16 - White blood cell count at week 16 (±4) of treatment; wbc_40 - White blood cell count at week 40 (±4) of treatment; IQR - interquartile range; SD - Standard deviation; Min - Minimum; Max - Maximum

Supplementary Table S2. Statistics for qualitative variables with grouping. The Chi-squared and Fischer's exact tests were used to determine associations between the MTX and BIOL groups for given variables (p-value).

Variable	Level	Missing (n)	n			% row		% column		% total		Association test	
			biol	mtx	total	Bio	mtx	Biol	mtx	biol	mtx	method	p
gender	M	0	123	29	152	80.92%	19.08%	56.16%	60.42%	46.07%	10.86%		
	W		96	19	115	83.48%	16.52%	43.84%	39.58%	35.96%	7.12%		
	Total		219	48	267							Chi-square	0.5900
type_of_drug	IL-12/23i	0	6	0	6	100.00%	0.00%	2.74%	0.00%	2.25%	0.00%		
	IL-17Ais		59	0	58	100.00%	0.00%	26.48%	0.00%	21.72%	0.00%		
	IL-17AFi		21	0	21	100.00%	0.00%	9.59%	0.00%	7.87%	0.00%		
	IL-23is		113	0	113	100.00%	0.00%	51.60%	0.00%	42.32%	0.00%		
	TNFis		20	0	20	100.00%	0.00%	9.13%	0.00%	7.49%	0.00%		
	MTX		0	48	48	0.00%	100.00%	0.00%	100.00%	0.00%	17.98%		
	Total		219	48	267							Fisher exact (RxC)	0.0000
drug	Adalimumab	0	18	0	18	100.00%	0.00%	8.22%	0.00%	6.74%	0.00%		
	Bimekizumab		22	0	22	100.00%	0.00%	10.05%	0.00%	8.24%	0.00%		
	Guselkumab		32	0	32	100.00%	0.00%	14.61%	0.00%	11.99%	0.00%		
	Infliximab		2	0	2	100.00%	0.00%	0.91%	0.00%	0.75%	0.00%		
	Ixekizumab		22	0	22	100.00%	0.00%	10.05%	0.00%	8.24%	0.00%		
	Risankizumab		70	0	70	100.00%	0.00%	31.96%	0.00%	26.22%	0.00%		
	Sekukinumab		36	0	36	100.00%	0.00%	16.44%	0.00%	13.48%	0.00%		
	Tildrakizumab		11	0	11	100.00%	0.00%	5.02%	0.00%	4.12%	0.00%		
	Ustekinumab		6	0	6	100.00%	0.00%	2.74%	0.00%	2.25%	0.00%		
	MTX		0	48	48	0.00%	100.00%	0.00%	100.00%	0.00%	17.98%		
	Total		219	48	267							Fisher exact (RxC)	0.0000
pasi_0	≤5	0	13	2	15	86.67%	13.33%	5.94%	4.17%	4.87%	0.75%		
	≥ 10		190	45	235	80.85%	19.15%	86.76%	93.75%	71.16%	16.85%		
	5--10		16	1	17	94.12%	5.88%	7.31%	2.08%	5.99%	0.37%		
	Total		219	48	267							Fisher exact (RxC)	0.4292
sp_loc	Ano-Gen	234	3	0	3	100.00%	0.00%	10.34%	0.00%	9.09%	0.00%		
	Head		16	0	16	100.00%	0.00%	55.17%	0.00%	48.48%	0.00%		
	Nail		7	0	7	100.00%	0.00%	24.14%	0.00%	21.21%	0.00%		
	PAL-FEET		3	4	7	42.86%	57.14%	10.34%	100.00%	9.09%	12.12%		

	Total		29	4	33							Fisher exact (RxC)	0.0024
pasi_16	≤5	2	98	12	110	89.09%	10.91%	44.75%	26.09%	36.98%	4.53%		
	≥ 10		11	9	20	55.00%	45.00%	5.02%	19.57%	4.15%	3.40%		
	0		98	7	105	93.33%	6.67%	44.75%	15.22%	36.98%	2.64%		
	5--10		12	18	30	40.00%	60.00%	5.48%	39.13%	4.53%	6.79%		
	Total		219	46	265							Fisher exact (RxC)	0.0000
pasi_40	≤5	66	64	10	74	86.49%	13.51%	36.16%	41.67%	31.84%	4.98%		
	≥ 10		16	4	20	80.00%	20.00%	9.04%	16.67%	7.96%	1.99%		
	0		94	3	97	96.91%	3.09%	53.11%	12.50%	46.77%	1.49%		
	5--10		3	7	10	30.00%	70.00%	1.69%	29.17%	1.49%	3.48%		
	Total		177	24	201							Fisher exact (RxC)	0.0000
withdrawal	AE	1	11	7	18	61.11%	38.89%	5.05%	14.58%	4.14%	2.63%		
	cont		158	27	185	85.41%	14.59%	72.48%	56.25%	59.40%	10.15%		
	InP		13	9	22	59.09%	40.91%	5.96%	18.75%	4.89%	3.38%		
	InS		36	5	41	87.80%	12.20%	16.51%	10.42%	13.53%	1.88%		
	Total		218	48	266							Fisher exact (RxC)	0.0023
CCI	0	0	96	18	114	84.21%	15.79%	43.84%	37.50%	35.96%	6.74%		
	1		50	12	62	80.65%	19.35%	22.83%	25.00%	18.73%	4.49%		
	2		46	9	55	83.64%	16.36%	21.00%	18.75%	17.23%	3.37%		
	3		15	2	17	88.24%	11.76%	6.85%	4.17%	5.62%	0.75%		
	4		6	2	8	75.00%	25.00%	2.74%	4.17%	2.25%	0.75%		
	5		6	1	7	85.71%	14.29%	2.74%	2.08%	2.25%	0.37%		
	6		0	1	1	0.00%	100.00%	0.00%	2.08%	0.00%	0.37%		
	8		0	1	1	0.00%	100.00%	0.00%	2.08%	0.00%	0.37%		
	9		0	1	1	0.00%	100.00%	0.00%	2.08%	0.00%	0.37%		
	10		0	1	1	0.00%	100.00%	0.00%	2.08%	0.00%	0.37%		
	Total		219	48	267							Fisher exact (RxC)	0.0537

AE - Adverse Effects; AoO- Age of onset; Ano-Gen - Anogenital area; BMI - Body Mass Index; CCI - Charlson Comorbidity Index; cont - continuation of treatment; IL-12/23i - Interleukin 12 and 23 inhibitor; IL-17Ais - Subunit A of interleukin 17 inhibitors; IL-17AFi - Subunit A and F of interleukin 17 inhibitor; IL-23is - Interleukin 23 inhibitors; TNFis - Tumor Necrosis Factor-alpha inhibitors; InP - Primary failure; InS - Secondary failure; MTX - Methotrexate; PAL-FEET - Palmoplantar localization of psoriatic lesions; PASI_0 - Baseline Psoriasis Area and Severity Index; PASI_4 - Psoriasis Area and Severity Index score at week 4 (±1) of biological treatment; PASI_16 - Psoriasis Area and Severity Index score at week 16 (±4) of biological treatment; PASI_40 - Psoriasis Area and Severity Index score at week 40 (±4) of biological treatment; p - p-value; sp_loc - Special locations; ToT - Time of treatment; M - men; W - women; BIOL - Biological drugs
